# Optimal forecasting accuracy using Lp-norm combination

**DOI:** 10.1007/s40300-021-00218-5

**Published:** 2021-08-07

**Authors:** Massimiliano Giacalone

**Affiliations:** Department of Economics and Statistics, University of Naples “Federico II”, Naples, Italy

**Keywords:** Lp-norm estimators, Generalized error distribution, Forecast combination, Financial time series, GARCH models

## Abstract

A well-known result in statistics is that a linear combination of two-point forecasts has a smaller Mean Square Error (MSE) than the two competing forecasts themselves (Bates and Granger in J Oper Res Soc 20(4):451–468, 1969). The only case in which no improvements are possible is when one of the single forecasts is already the optimal one in terms of MSE. The kinds of combination methods are various, ranging from the simple average (SA) to more robust methods such as the one based on median or Trimmed Average (TA) or Least Absolute Deviations or optimization techniques (Stock and Watson in J Forecast 23(6):405–430, 2004). Standard regression-based combination approaches may fail to get a realistic result if the forecasts show high collinearity in several situations or the data distribution is not Gaussian. Therefore, we propose a forecast combination method based on Lp-norm estimators. These estimators are based on the Generalized Error Distribution, which is a generalization of the Gaussian distribution, and they can be used to solve the cases of multicollinearity and non-Gaussianity. In order to demonstrate the potential of Lp-norms, we conducted a simulated and an empirical study, comparing its performance with other standard-regression combination approaches. We carried out the simulation study with different values of the autoregressive parameter, by alternating heteroskedasticity and homoskedasticity. On the other hand, the real data application is based on the daily Bitfinex historical series of bitcoins (2014–2020) and the 25 historical series relating to companies included in the Dow Jonson, were subsequently considered. We showed that, by combining different GARCH and the ARIMA models, assuming both Gaussian and non-Gaussian distributions, the Lp-norm scheme improves the forecasting accuracy with respect to other regression-based combination procedures.

## Introduction

Model instabilities are deeply rooted in real-life forecasting challenges because models are uncertain and mutable. Moreover, in statistics, the Data Generating Process (DGP), refers to the true process which generates the data and it is different from a model of the data. Indeed, statistical models could be either mispecified or incomplete [53].

This is a fact that is widely accepted in theory, but less widely applied in practice in the field of statistics, in which researchers often still hang on to the conceptual error of assuming one true data-generating process and focussing too much on model selection in order to find the one true model.

In this respect, Hansen [26] takes note of this and related misconceptions of econometric forecasting practice in his essay on the challenges for statistical model selection.

An obvious alternative to choosing a single ‘best’ forecasting method is to combine forecasts from different models. The combination could be achieved in different ways. One of the most common is by regression approaches based on Ordinary Least Squares (OLS) estimation technique. Below, we propose an alternative and more general approach for combining models and/or forecasts, based on Lp-norm estimators [40].

In this paper we propose to study the effectiveness of Lp-norm estimators in combining volatility forecast. We consider the volatility of stock market data because they empirically show non-Gaussian distributions. Indeed the Lp-norm should work better than OLS (as it is) in presence of non-Gaussian forecasts. The GARCH distributions play an important role in the volatility measurement and in the value-at-risks topics [10].

The main motive of this paper is to measure the performance of GARCH techniques for forecasting combination by using different distribution model. The different GARCH distribution models present in the paper are the t-student, the Gaussian, the GED jointly considered with some ARMA models. We try to show the advantages of GED GARCH over the classical methods, for example, the t-student GARCH and the Gaussian GARCH.

The paper is, then, structured as follows. In the next section, we will present an historical survey about the literature of forecast combination and we will glance at some of the main methods of combination since its introduction. We will also briefly introduce our method. In Sect. 3, we introduce the main recent methods for better achieve the forecast combination and we show the advantages and the disadvantages of the different combination approaches.

In Sect. 4, we bring up for discussion the Generalized Error Distributions (G.E.D.) explaining in detail our approach based on Lp-norm estimators by proposing a new algorithm to combine the forecasts. Paragraph 4 shows, instead some comparative Montecarlo experiments where the overperformance of the proposed methodology is presented in the situations of multicollinearity and non-Gaussianity by means of a simulation study considering heteroskedastic and homoskedastic innovations.

The real data application is proposed, now, in Sect. 6, in which we attempt to determine if the results obtained in the simulation study are possible also in reality. In the last paragraph, some final comments and observations are offered.

Finally, in the appendix A and B we show the commented R studio scripts that we built and used to estimate the forecast and the combination in the simulated and the empirical study, whilst in appendix C some graphical findings of the simulation study are reported.

## The main approaches to combine forecast in models: a review

The common way of choosing a forecasting model is to consider the one which allows to obtain the most accurate forecasts, discarding the others. This method has been re-evaluated since the introduction of forecast combinations, which integrate the information generated by multiple forecasts and using it to get a more accurate model. Combining forecasts is a useful practice as the excluded models may contain important information that is not present in the individual forecasts.

When the forecasts diverge, due to the assumptions made in the forecasting model (linearity, type of distribution, the variability of the parameters, etc.), the decision-maker who wants a single forecast faces a thorny problem of choice. Forecast combination constitutes a solution to this problem and refines the forecast. This is still true almost 30 years later and it is called the “forecast combination puzzle”, a term coined by Stock and Watson [47]. Hibon and Evgeniou [29] verified that the linear combination, using the individually worst forecasts, still leads to better results than individual ones. Hence, choosing an individual method out of a set of available methods is riskier than choosing a combination.

First of all, let' s review the main forecast combination methodologies introduced since the seminal paper by Bates and Granger [7]. In the last few decades, several methodologies have been put forward both in the theoretical and empirical literature. Andrawis et al. [1] even suggest using hierarchical forecast combinations, i.e. combining joining forecasts. Our purpose is not to investigate the best combination method by comparing all the ones proposed in the literature. A lot depends on the specifics of the data available and the differences in modelling approaches. Rather, we provide a new way of combining within regression framework which works well in several real-life situations as non-Gaussian forecast density or collinear forecasts.

The Bayesian approach was suggested by Min and Zellner [37] which consider various time series models, that produce forecasts of production growth rates for 18 countries over a 13-year period to combine models and their forecasts. Optimal Bayesian combination procedures consider the use of rear dimensions for alternative models and are also used in predictive testing to decide whether or not to combine alternative model forecasts. Applying Bayesian pooling techniques it turns out that it is not always optimal to combine forecasts.

A well-known result is that a more accurate forecast can be obtained by making a linear combination of two-point predictions. The combination allows to improve the forecast, which will have a smaller mean square error (MSE) than the two competing forecasts. Therefore, by combining multiple forecasts, decision-making becomes simpler. The only case in which no improvements are possible is when one of the single forecasts is already the optimal one in terms of MSE [30].

A simple procedure to combine forecasts is to take an arithmetic average of the single forecasts that serves as a useful benchmark. Given a forecast $\widehat{{y}_{i}}$ provided by the i-th forecasting model (or from the i-th expert forecaster), the well-known results is that the Mean Square Error of the combination of all $\widehat{{y}_{i}}$ (e.g. $\stackrel{-}{y}$) is lower than the one of $\widehat{{y}_{i}}$ itself:

$$ MSE_{{\widehat{{y_{i} }}}}  > MSE_{{\mathop y\limits^{ - } }}\quad  {}\forall i $$

In a paper from 1963, Barnard showed an empirical example in which an arithmetic average of two forecasts had a smaller Mean Square Error (MSE) than either of the individual forecasts. If the component forecasts are not biased, combining with mean is a good method. Clemen [15] summarized the literature on forecast combinations and concluded that combining forecasts of various economic and financial variables led to increased forecast accuracy. He states that using a combination of forecasts is equivalent to admitting that the meteorologist is unable to construct a specified model and trying to combine increasingly elaborate models is incorrect. Similar conclusions were reached by Aksu and Gunter [4] based on macroeconomic variables and firm‐specific series, by Makridakis and Hibon [36] based on the so‐called M3 competition, by Stock and Watson [46, 47] across various economic and financial variables, by Swanson and Zeng [48] using US macroeconomic variables [2, 14, 15] deemed that this equal weighting of component forecasts is often the best strategy.

According to Armstrong [2] it is necessary to combine predictions from essentially different methods that are derived from different sources. In particular, in order to improve the accuracy of forecasts, it has been suggested using formal procedures to combine forecasts.

Another simple and appealing combination method is using the median of the component forecasts. The median is insensitive to outliers, which can be relevant for some application. Palm and Zellner [41], Ruiz and Nieto [43] Hendry and Clements [28] suggested that simply averaging may not be a suitable combination method when some of the component forecasts are biased.

Jose and Winkler [33] proposed the usage of the trimmed mean approach instead of a simple average or median combination. Sometimes, the simpler methods, such as median [42], can generate satisfactory results compared to more complex methods.

It might be better to combine the forecasts using more sophisticated rules: one of them is by Least Squares regression. The idea to use regression for combining forecasts was put forward by Crane and Crotty [16] and successfully driven to the forefront by Granger and Ramanathan [25]. Using this approach, the combined forecast is a linear function of the individual forecasts where the weights are determined using a regression of the individual forecasts on the target itself:

1 $$y=\alpha +\sum _{i=1}^{p}{w}_{i}{f}_{i}+\varepsilon ,$$

An advantage of the OLS forecast combinations is that the combined forecast is unbiased, even if one of the individual forecasts is biased. A disadvantage is that the method does not restrict the combination weights (they do not add up to one and can be negative). In this respect, the Constrained Least Squares (CLS) regression, could be used to get the weights of the forecast, determined by minimizing the squared errors under the constraint of non-negativity, sum one and intercept equal to zero. This method, due to the absence of the intercept, might lead to a distorted forecast if one of the individual forecasts is biased. As a consequence, CLS does not always achieve the best level of accuracy in terms of MSE.

While the OLS regression estimates the coefficients by minimizing the sum of squared errors, we may want to estimate those coefficients differently [18, 27], minimizing a different loss function, for example, the absolute sum of squares (LAD). The Least Absolute Deviation method has two main advantages: first of all, this method has a lower sensitivity to outliers. Secondly, when predictors are highly correlated, it performs better. This suggests that LAD combination should be preferred to OLS in these situations [52].

Dielman [17] proposed a Monte Carlo simulation to evaluate predictions by comparing the method of the minimum absolute value with the regression equations estimated by the least squares method. In the presence of outliers, the absolute minimum value forecasts were higher and performed better than forecasts obtained by the least squares method. These results underline the importance of using absolute regression of the minimum value (or some other robust regression method) in the presence of outliers.

However, we could potentially still do better by providing a more general framework for combining forecasts through the Lp-norm estimators. As we will see more in detail, Lp-norm is generic, whereas LAD and Least Squares are special cases. When forecasts are non- Gaussian (e.g. volatility forecasts in financial markets) or collinear, OLS is no longer the best way of estimating quantities since it becomes less efficient as its variance increases. Another noticeable limitation occurs when the density function of the forecasts is not Gaussian distributed since by using a different estimator we gain in efficiency.

In this paper we propose a forecast combination method based on Lp-norm estimator, where the minimization of residuals is done according to estimated data kurtosis and the selection of more relevant forecast is achieved through some procedures proposed in literature. Among them the Lp-med combined method is a recent proposal to estimate *p *[23].

If the errors follow a Gaussian distribution, the forecast obtained with a linear combination of forecasts using Lp-norm parameters (L_2_ norm in this case) gives satisfying results, the mean square error is slightly higher than the MSE using the OLS parameters but lower than the other methods (constrained least squares, least absolute deviation, generalized least squares, etc.).

Lp-norm estimators are useful generalizations of Ordinary Least Squares estimators, based on the Generalized Error Distribution (GED) [21].

Below, we introduce a table in which we provide a summary of the state of the art about the best-known methods of forecast combination and the main authors to whom they refer (Table 1).

**Table 1:** Summary of the main methods of forecast combination

Forecast combination methodology	Main content	Main authors and references
Arithmetic average	A simple average of two forecasts has a lower mean squared error	Barnard *(Journal of the Royal Statistical Society, 1963)*
Optimal variance minimizing weight	In general, a linear combination of two forecasts have a smaller MSE	Bates and Granger *(Journal of the Operational Research Society, 1969)*
Constrained Least Squares	Regression with OLS method under the constraint that the weights are non-negative	Granger and Ramanathan *(Journal of Forecasting, 1984)*
Bayesian Model Averaging	Combination using posterior odds ratios derived by fixed and time varying parameter models	Min and Zellner *(Journal of Econometrics, 1993)*
Ordinary Least Squares	Weight estimation using the OLS method and performing a regression of the objective forecast	Granger and Ramanathan *(Journal of Forecasting, 1984)*
Least Absolute Deviation (LAD)	Computes forecast combination weights using Least Absolution Deviation regression	Dielman *(Journal of Forecasting, 1986)*
Trimmed mean	Remove a percentage of outliers when using at least 5 forecasts to reduce the error component	Armstrong *(Principles of Forecasting, 2001)*
Median	Use the median of the component forecasts, could be relevant because it is insensitive to outliers	Stock and Watson *(Journal of Forecasting, 2004)*
Principal component forecast combination	Regression of the principal components gained from the common factors of the forecasts	Stock and Watson *(Journal of Forecasting, 2004)*
Lp-norm estimators	Generalization of OLS combination method particularly used in case of collinear and/or non-Gaussian data	Giacalone, Panarello and Mattera (*Quality & Quantity, 2018*)

Source: own elaboration

## Combine or not combine: recent contributions

The search for new forecast combination methods is still very active. Even in recent years new methods are experienced and older methods are tested such as the approach of forecast combination method for the M4-competition given by Jaganathan and Prakash [31].

Atiya [3] has provided a brief analysis of the reasons why forecast combinations are successful. There are many other very insightful works in the literature on this important topic that considers several different aspects, such as the effects of serial correlation, heteroskedasticity, structural breaks, estimation error in the combination weights.

Xiao et al. [54] stated that the combination models demonstrate better performance than the individual forecast models do. The most common procedure for combining forecasts is to assign a weight to each model based on its past forecast performance and then aggregate the related weighted forecasts. At the same time, there are other forecasting models, for example, methodologies that are based on the use of intelligent optimization algorithms to select or optimize the parameters of statistical models in the training phase. Another class of combined models is that which involves processing the time series by breaking it down into more stationary and regular sub-series that are easier to identify and deal with, which aim is to apply an adequate forecast model for each decomposed series and finally, to aggregate the related forecasts. Finally, all those models, which we will call hybrid models, are also combined models, in which the forecast of the historical series is carried out, also taking into account the forecast of the residuals resulting from the main model applied to the original series.

Wang et al. [51] in their study, forecast the realized volatility of the S&P 500 index using the heterogeneous autoregressive model for realized volatility (HAR-RV) and its various extensions.

Models under consideration take into account the time-varying property of the models’ parameters and the volatility of realized volatility. A dynamic model averaging (DMA) approach is used to combine the forecasts of the individual models. Empirical results of their paper suggest that DMA can generate more accurate forecasts than individual model in both statistical and economic senses. Models that use time-varying parameters have greater forecasting accuracy than models that use the constant coefficients.

Zhang et al. [56], to improve the forecasting accuracy, proposed a new hybrid model based on improved empirical mode decomposition (IEMD), autoregressive integrated moving average (ARIMA) and wavelet neural network (WNN) optimized by fruit fly optimization algorithm (FOA) is proposed and compared with some other models. Zhang et al. [57] employed two prevailing shrinkage methods, the elastic net and lasso in the prediction of oil price. Their findings suggests that the elastic net and lasso exhibit significantly better out-of-sample forecasting performance than not only the individual HAR-RV-type models but also the combinations approaches. The elastic net and lasso also exhibit higher directional accuracy and yield sizeable economic gains for asset allocation. Liang et al. [34] investigated which uncertainty indices have predictability for oil price by employing the standard predictive regression framework, various model combination and two prevailing model shrinkage methods (Elastic net and Lasso). The authors analyzing the two model shrinkage methods, namely Elastic net and Lasso, outperform other individual and combination models in forecasting the crude oil market volatilities.

Forecast combinations, while appealing in theory, are at a disadvantage over a single forecast model because they introduce parameter estimation error in cases where the combination weights need to be estimated. Finite sample errors in the estimates of the combination weights can lead to poor performance of combination schemes that dominate in large samples. Is better combine or not to combine the forecasts or rather simply attempt to identify the single best forecasting model? When the information sets are unobserved it is often justified to combine forecasts provided that the private (non-overlapping) parts of the information sets are sufficiently important. When forecast users do have access to the full information set used to construct the individual forecasts combinations may be less justified. Finding a ‘best’ model may of course be rather difficult if the space of models included in the search is high dimensional and the time-series short [50].

In situations where the data is not very informative and it is not possible to identify a single dominant model, it makes sense to combine forecasts. The Schwarz Information Criteria (SIC) can be used as a great alternative for choosing which subset of predictions to combine. Swanson and Zeng [48] observed that combination forecasts from a simple averaging approach MSE‐dominate SIC combination forecasts less than 25% of the time in most cases, while other ‘standard’ combination approaches fare even worse. Estimation errors in the combination weights tend to be particularly large due to difficulties in precisely estimating the covariance matrix. One answer to this problem is to simply ignore correlations across forecast errors. Stock and Watson [45] propose a broader set of combination weights that also ignore correlations between forecast errors but base the combination weights on the models’ relative MSE performance raised to various powers.

In conclusion, although the results got in the previous paragraphs show always that the forecasts combination has a MSE smaller than the values of the single forecasts, it is necessary to properly evaluate in which situations it is convenient to combine the single predictions and in which situations it is not convenient (Table 2).

**Table 2:** Vantages and disadvantages of different approaches

Approaches to combine	Description	Advantages	Disadvantages
Based on weighting coefficients	To each model is assigned a weight based on its forecasting capacity	They are easy to implement, they adapt quickly to new data available	They do not guarantee the best forecast along the entire forecast horizon, they require an additional model for determine the weights
Based on pre-processing techniques some data	The series is decomposed into sub-series and the relative predictions are made	Many examples are available in the literature, guaranteeing a high performance	They require in-depth mathematical knowledge of decomposition methods; they slowly adapt to new data
Based on parameter selection and optimization techniques	The parameters of a forecast model are optimized	Many examples are available in the literature, the configuration is relatively basic	They are difficult to program; they require a lot of effort computational
Based on hybrid models	A forecast model is also built for the residuals generated by the main forecast model applied to the time series	They ensure a reduction in systematic error and therefore a high accuracy	They are inefficient from the point of view of computation time

Source: own elaboration

## The proposed methodology

The Generalized Error Distribution (GED), also known as Exponential Power Function (EPF), is a family of probability functions proposed by Subbotin [49] and Mineo [38] that generalize the Gaussian distribution. An asymmetrical version of the Gaussian distribution has also been proposed in the literature,seen among others [10, 11].

The GED constitutes a valid generalization of the Gaussian distribution that is usually assumed, even though, depending on the available data, it is not always fully supportable.

It constitutes a parametric alternative to previous robust methods [39].

The density function of the GED is:

2 $$f\left(x{|\mu }_{p},{\sigma }_{p},p\right)=\frac{p}{2{\sigma }_{p}}{{\Gamma }\left(\frac{1}{p}\right)}^{-1}\mathrm{e}\mathrm{x}\mathrm{p}\left\{-{\left|\frac{x-{\mu }_{p}}{{\sigma }_{p}}\right|}^{p}\right\}$$

where ${\mu }_{p}\in (-\infty ,+\infty )$ is the location parameter, ${\sigma }_{p}$ is the scale parameter, that is positive with ${\sigma }_{p}\in (0,+\infty )$ and the shape parameter $p$ is a measure of fatness of tails where $p\in (0,+\infty )$ and $x\in \mathbb{R}$ [58].

Considering the Pearson kurtosis index ${\beta }_{2}$, we distinguish:

- 0 < $p$  < 1: double exponential distribution, ${\beta }_{2}$ > 6;
- 1 < $$p$$  < 2: leptokurtic distribution, 3 < $${\beta }_{2}$$< 6;
- $$p$$ > 2: platikurtic distribution, 1.8 <  $${\beta }_{2}$$ < 3.
  For specific values of *p*, we have:
- the Laplace distribution ($p$ = 1, ${\beta }_{2}$ = 6);
- the Gaussian distribution ($p$ = 2, ${\beta }_{2}$ = 3);
- the Uniform distribution ($p$ —>  ∞, ${\beta }_{2}$ = 1.8).

**Fig. 1:**
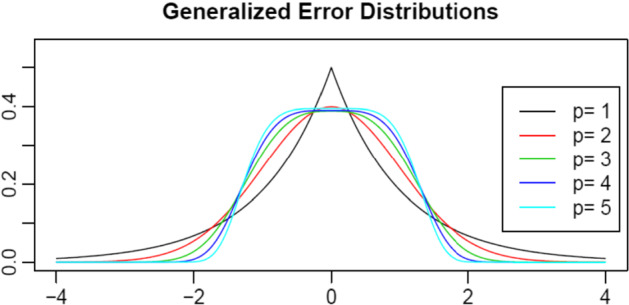
Generalized Error Distribution probability density functions with different shape parameters

Considering a sample of *n* observed data (${x}_{i}$, ${y}_{i}$), a general linear regression model is (Fig. 1):

3 $${y}_{i}=g\left({x}_{i},\theta \right)+{\varepsilon}_{i}$$

With g(·) linear function.

Lp-norm estimators are useful generalizations of ordinary least squares estimators, obtained by replacing the exponent 2 with a general exponent $p$; similarly, we obtain the LAD (Least Absolute Deviation) with p = 1. Therefore, they minimize the sum of the p-th power of the absolute deviations of the observed points from the regression function [23]:

4 $$ \begin{aligned}    & S_{p} \left( \theta  \right) = {\mkern 1mu} \sum\limits_{{i = 1}}^{n} | y_{i}  - g(x_{i} ,\theta )|^{p}  \\     & \quad {\text{with}}\;1 \le {\mkern 1mu} p < \infty  \\  \end{aligned}  $$

where $$x={y}_{i}$$; $${\mu }_{p}=g({x}_{i},\theta )$$

Under the regular assumptions, the log-likelihood associated with the sample is given by:

5 $$ l\left( {\theta ,\, \sigma _{p} ,\, p} \right) = n{\text{og}}\left[ {2p^{{1/p}} \sigma _{p} \Gamma (1 + 1/{\text{p}})} \right] - \, \left( {\left. {p\sigma _{p}^{p} } \right)^{{ - 1}} } \right.\sum\limits_{{i = 1}}^{n} {\left| {y_{i} \left. { - g\left( {x_{i} ,\, \left. \theta  \right)} \right.} \right|^{p} } \right.}  $$

6 $$ \frac{{\partial l}}{{\begin{array}{*{20}c}    {\partial \theta _{j} }  \\   \end{array} }} = \sum\limits_{{i = 1}}^{n} {\left| {y_{i} \left. { - g\left( {x_{i} ,\, \left. \theta  \right)} \right.} \right|^{{p - 1}} } \right.} sign\left( {y_{i}  - g\left( {x_{i} ,\, \theta } \right)} \right)\frac{{\partial g}}{{\partial \theta _{j} }} = 0 $$

7 $$ \sum\limits_{{i = 1}}^{n} {\left| {y_{i} \left. { - g\left( {x_{i} ,\, \left. \theta  \right)} \right.} \right|^{p} } \right.}  = {\text{min}}\quad with\, p \ge 1{\text{ and }}1 \le j < m; $$

The result of the last equation shows that maximum likelihood estimators (we considered a model with *m* parameters) are equivalent to the Lp-norm estimators if the value of $p$ is known. If *p* is unknown, we have the problem to estimate it.

The GED assumption has been applied with success also in forecasting literature. A clear example is represented by the GED-GARCH model for predicting volatility [32].

Indeed, since the introduction of Generalized Autoregressive Conditional Heteroskedastic (GARCH) model of Bollerslev [9], thousands of articles have been published applying the model on financial series. Because of many observed phenomena do not exhibit Gaussian law, therefore we extend the classical econometric models to the case with non-Gaussian distribution. As the extension we propose to use the Generalized Error Distribution that it has often revealed itself one of the most adequate to examined time series in the literature.

In this paper we illustrate the main properties of the considered models and present testing and estimation procedures. We illustrate the theoretical results with real financial data analysis and simulation studies.

The Normal distribution is the usual assumption in any time series estimation, but due to the fact that the distribution of GARCH process is leptokurtic, Normal distribution was found to be inappropriate in capturing the tail behavior of the series [55].

Even in the classical definition, it is assumed that the standardised residuals ${\varepsilon }_{t}$ of ARCH and GARCH are Gaussian random variables; in practice, it turns out that this assumption weakly corresponds to reality. An example of GED distribution in forecasting is the GED-GARCH model.

Since the Gaussian distribution is a special case of the GED the standard GARCH model is a particular case of the GED-GARCH. This means, in other words, that a GED-GARCH model takes the following form for the mean equation:

8 $${r}_{t}={u}_{t}+{a}_{t}$$

where

9 $${a}_{t}={\sigma}_{t}{\varepsilon }_{t}\quad {\varepsilon}_{t}\sim GED\left(\mu,\lambda,\zeta \right)$$

${u}_{t}$ is called conditional mean and is also expressed as:

$${\mu }_{t}=E\left({r}_{t}|{r}_{t-1},{r}_{t-2},\dots \right)$$

The conditional variance, on the other hand, can be expressed as follows:

$${\sigma }_{t}^{2}=E\left[{\left({r}_{t}-{\mu }_{t}\right)}^{2}|{\left({r}_{t-1}-{\mu }_{t}\right)}^{2},{\left({r}_{t-2}-{\mu }_{t}\right)}^{2},\dots \right]=E\left({{a}_{t}}^{2}|{{a}_{t-1}}^{2},{{a}_{t-2}}^{2},\dots \right)$$

where $\mu \in (-\infty ,+\infty )$, $\lambda$ is positive therefore $\lambda \in (0,+\infty )$ and also $\zeta$, which is a measure of fatness of tails is $\zeta \in (0,+\infty )$ and $x\in \mathbb{R}$.

However, the proposed parameterization is very used, especially in the GARCH models, and, precisely, we will develop the calculation of the parameters of the models for volatility on that one, assuming that ${\varepsilon }_{t}$ it follows a Generalized Error Distribution.

In fact, the parameterization proposed by Forbes et al. [19] is given by:

10 $$ f\left( {x;\mu ,\lambda ,\kappa } \right) = \frac{{exp\left( { - \frac{1}{2}\left| {\frac{{x - \mu }}{\lambda }} \right|^{{\frac{1}{\kappa }}} } \right)}}{{2^{{\kappa  + 1}} \lambda \Gamma \left( {\kappa  + 1} \right)}} $$

Or, with reparameterization: $\zeta =\frac{1}{\kappa }$ we can assume:

11 $$f\left(x;\mu ,\lambda ,\zeta \right)=\frac{aexp\left(-\frac{1}{2}{\left|\frac{t-\mu }{\lambda }\right|}^{\zeta }\right)}{{2}^{1+\frac{1}{\zeta }}\lambda {\Gamma }\left(\frac{1}{\zeta }+1\right)}$$

where $\zeta$ is the shape parameter (the equivalent of *p*), the scale parameter $\lambda$ is the equivalent of ${\sigma }_{p}$ and the last parameter $\mu$ that is ${\mu }_{p}$. Equation (11) is the parameterization from which we will start to estimate the parameters of the GED-GARCH model.

The algorithm used for real data application runs as follows. Given y_t_ a time series, we consider the following steps:

Step 0: For each time series y_t_ in the sample, we compute its descriptive statistics;
Step 1: We assess the time series stationarity by means of the Dickey–Fuller and KPSS unit root tests and use the first difference if the hypothesis of unit root is not rejected;
Step 2: We divide the sample into a training set and a testing set;
Step 3: We fit the following statistical models in training set: the ARMA(p,q) model, the Gaussian GARCH(p,q), the t-student GARCH(p,q), the GED GARCH(p,q);
Step 4: We estimate the forecasts with these single models and we calculated the Root Mean Square Error (MSFE);
Step 5: We combined these models with different forecast combination methods: Ordinary Least Squares (OLS), Constrained Least Squares (CLS), Least Absolute Deviation (LAD), Lp-norm;
Step 6: We computed: MSFE, Mean Error (ME), Mean Absolute Error (MAE), Mean Percentage Error (MPE), Mean Absolute Percentage Error (MAPE);
Step 7: We calculated the Q-Q plot for Gaussian GARCH and GED GARCH and we tested with the Jarque–Bera test both for evaluating the Gaussian normality of the data distribution.

## A comparative simulation study

Here is a short outline of what we are going to analyze in this paragraph. We have made twenty-four simulations: twelve with an AR(1) process and homoskedastic innovations and twelve with the same process but heteroskedastic innovations. In both cases we suppose three different shape parameters of the GED distribution (p = 1.5, in the case of leptokurtic distributions, p = 2, in the case of Gaussian distributions, p = 2.5, in the case of platikurtic distributions) and for each of them we suppose four different autoregressive parameters (φ = 0.2, low; φ = 0.4, medium–low; φ = 0.6, medium–high; φ = 0.8 high). We aim to show that in the presence of non-Gaussian data, combining forecasts with Lp-norm instead of OLS is a good choice since it is always better than LAD and CLS and sometimes better than OLS.

Hence, firstly we simulated 1000 historical series with 200 observations from an autoregressive process of order 1 or, in other words, an AR(1) with non-Gaussian innovation $${\varepsilon }_{t}$$ and autoregressive parameter $$\varphi $$. We assumed a Generalized Error Distribution G.E.D. with mean 0, scale parameter 1 and true shape parameter $$p$$. Then we split the overall sample in the training set $${(y}_{t}^{TRAIN}$$) and testing set ($${y}_{t}^{TEST})$$. With $${y}_{t}^{TRAIN}$$ (80% the of observations) we ran the model to obtain 40 forecasts $${y}_{t}^{FOR}$$. We used the remaining last 40 values $${y}_{t}^{TEST}$$ (the 20% of observations) to assess the accuracy of the model comparing $${y}_{t}^{FOR}$$ with the true $${y}_{t}^{TEST}$$ [22]. The mean squared forecast errors are computed as the average value of 1000 replications [12].

In the Tables 3, 4 and 5 we illustrate the case of homoskedastic innovation.

**Table 3:** Mean Squared Forecast Errors for different method of combination models: Homoskedastic cases (*p* = 1.5)

p = 1.5	Mean Squared Forecast Errors			
	Phi = 0.2	Phi = 0.4	Phi = 0.6	Phi = 0.8
ARMA (1,1)	1.153137	1.239956	1.423515	1.984615
GARCH (1,1)	1.153425	1.239805	**1.422018**	1.991198
t-GARCH (1,1)	**1.152806**	1.239714	1.422944	1.981163
GED-GARCH (1,1)	1.153383	**1.239546**	1.423393	**1.981098**
OLS	1.049017	1.093995	1.164632	1.223049
CLS	1.137343	1.214243	1.383432	1.813251
LAD	1.058551	1.109501	1.185866	1.254626
Lp-norm	**1.045238**	**1.090459**	**1.159404**	**1.211461**

Bold font highlights the models with the lowest MSFE for either single models and alternative combination schemes

**Table 4:** Mean Squared Forecast Errors for different method of combination models: Homoskedastic cases (*p* = 2)

p = 2	Mean Squared Forecast Errors			
	Phi = 0.2	Phi = 0.4	Phi = 0.6	Phi = 0.8
ARMA (1,1)	1.027575	**1.100533**	1.292379	**1.717079**
GARCH (1,1)	1.027191	1.101001	1.288842	1.719956
t-GARCH (1,1)	1.027294	1.101263	**1.287817**	1.719557
GED-GARCH (1,1)	**1.027432**	1.101095	1.290064	1.717697
OLS	0.943194	0.975322	1.031283	1.084871
CLS	1.018574	1.081655	1.231272	1.587086
LAD	0.952778	0.989847	1.048591	1.113149
Lp-norm	**0.939177**	**0.971166**	**1.021963**	**1.073453**

Bold font highlights the models with the lowest MSFE for either single models and alternative combination schemes

**Table 5:** Mean Squared Forecast Errors for different method of combination models: Homoskedastic cases (*p* = 2.5)

p = 2.5	**Mean Squared Forecast Errors**			
	**Phi = 0.2**	**Phi = 0.4**	**Phi = 0.6**	**Phi = 0.8**
ARMA (1,1)	0.956661	1.020648	1.185063	**1.619036**
GARCH (1,1)	**0.956209**	**1.020535**	1.184893	1.630448
t-GARCH (1,1)	0.956311	1.020558	**1.184213**	1.624769
GED-GARCH (1,1)	0.956220	1.020726	1.186083	1.635494
OLS	0.875576	0.898749	0.960902	1.009944
CLS	0.947621	1.001617	1.147665	1.481237
LAD	0.884855	0.915376	0.978349	1.027941
Lp-norm	**0.872641**	**0.897653**	**0.955601**	**0.993774**

Bold font highlights the models with the lowest MSFE for either single models and alternative combination schemes

As we can see from the Table 3 with the case *p* = 1.5 (platikurtic case) with homoskedastic innovations, we have estimated the models ARMA (1,1), GARCH (1,1), t-GARCH (1,1) and GED-GARCH (1,1).

From the analysis of Table 3, we can see that the best model (i.e. the model with the lowest MSFE) is the GED-GARCH (1,1) because it has the lowest MSFE either for phi = 0.4 or for phi = 0.8. Moreover we combine the forecasts with the OLS, the CLS, the LAD and the Lp-norm combination methods. We know that combining forecasts increases the accuracy and, as proof of this, the MSFE of the single forecasts are always greater than the MSFE of the forecast combinations [13]. The best combination method is the Lp-norm, indeed it has the smallest MSFE for all the considered phi parameters.

In Table 4, we show the case *p* = 2 (Gaussianity) with homoskedastic errors, where we estimated the same models as before. In that case the ARMA (1,1) is the best model, because it has the lowest loss function value (MSFE) for phi = 0.4 and for phi = 0.8. We know that when the shape parameter *p*, is equal to 2, the GED distribution is equivalent to the Gaussian distribution. The most accurate combination method to combine forecasts is the Lp-norm but, as we can see, the MSFE are very close to the forecast combination values obtained by OLS method.

From Table 5, we can observed that the best forecasting model is the GARCH in the cases p = 2.5 Phi = 0.2 and Phi = 0.4. For Phi = 0.6 the best model is the t-GARCH (1,1) whilst for Phi = 0.8 the best forecast model is the ARMA (1,1). Again in the case of *p* = 2.5 (leptokurtic cases) the Lp-norm provides the most accurate forecast combination for any values of Phi and, once again, the best forecast combination method is the Lp-norm.

In the following Tables 6, 7 and 8, we illustrate the case of heteroschedastic innovations.

**Table 6:** Mean Squared Forecast Errors for different method of combination models: heteroschedastic cases (*p* = 1.5)

p = 1.5	Mean Squared Forecast Errors			
	Phi = 0.2	Phi = 0.4	Phi = 0.6	Phi = 0.8
ARMA (1,1)	0.2923143	0.2671772	0.3309744	0.5162154
GARCH (1,1)	**0.2868437**	0.2663302	0.3247703	0.5453012
t-GARCH (1,1)	0.2869097	**0.2659165**	0.3248029	**0.5052075**
GED-GARCH (1,1)	0.2868528	0.2663975	**0.3247310**	0.5258339
OLS	0.2070518	**0.2060281**	**0.2271676**	0.2313181
CLS	0.2238759	0.2272992	0.2647644	0.3246229
LAD	0.2042246	0.2110616	0.2340576	0.2405602
Lp-norm	**0.2015794**	0.2068880	0.2273586	**0.2308171**

Bold font highlights the models with the lowest MSFE for either single models and alternative combination schemes

**Table 7:** Mean Squared Forecast Errors for different method of combination models: heteroschedastic cases (*p* = 2)

p = 2	Mean Squared Forecast Errors			
	Phi = 0.2	Phi = 0.4	Phi = 0.6	Phi = 0.8
ARMA (1,1)	0.2655602	0.3146837	0.3230243	0.4639478
GARCH (1,1)	**0.2647194**	**0.3120864**	**0.3216893**	0.4797646
t-GARCH (1,1)	0.2648118	0.3121706	0.3217895	**0.4406607**
GED-GARCH (1,1)	0.2647282	0.3121699	0.3217994	0.4512571
OLS	**0.2019596**	**0.2185927**	0.2211455	0.2290417
CLS	0.2181187	0.2428808	0.2596338	0.3332707
LAD	0.2046953	0.2233323	0.2248226	0.2387868
Lp-norm	0.2019741	0.2190896	**0.2185642**	**0.2282186**

Bold font highlights the models with the lowest MSFE for either single models and alternative combination schemes

**Table 8:** Mean Squared Forecast Errors for different method of combination models: heteroschedastic cases (*p* = 2.5)

p = 2.5	Mean Squared Forecast Errors			
	Phi = 0.2	Phi = 0.4	Phi = 0.6	Phi = 0.8
ARMA (1,1)	0.2728182	0.3582986	0.3373179	**0.5524382**
GARCH (1,1)	**0.2722719**	**0.3569198**	**0.3285978**	0.5579127
t-GARCH (1,1)	0.2722835	0.3571036	0.3286418	0.5382607
GED-GARCH (1,1)	0.2723097	0.3571202	0.3286167	0.5386678
OLS	**0.1972401**	0.2196049	0.2213709	**0.2319335**
CLS	0.2140279	0.2426766	0.2613526	0.3370463
LAD	0.2042953	0.2240118	0.2266377	0.2464403
Lp-norm	0.2011514	**0.2193382**	**0.2208288**	0.2364978

Bold font highlights the models with the lowest MSFE for either single models and alternative combination schemes

In th Table 6 with the case *p* = 1.5 (platikurtic case) with heteroskedastic innovations, we have estimated, again, the models ARMA (1,1), GARCH (1,1), t-GARCH (1,1) and GED-GARCH (1,1). From this table we can see that the best model (i.e. the model with the lowest MSFE) is the t-GARCH (1,1) because it has the lowest MSFE either for phi = 0.4 or for phi = 0.8.

Indeed we combine the forecasts with the OLS, the CLS, the LAD and the Lp-norm combination methods. We know that combining forecasts increases the accuracy. The most interesting thing is that the Lp-norm method is not the best forecast combination anymore, or better, it is the best for phi = 0.2 and for phi = 0.8 but for phi = 0.4 and for phi = 0.6 the OLS forecast combination method is the best one. Infact for phi = 0.4 and for phi = 0.6 the OLS Mean Squared Forecast Errors present the lowest values respect to all the considered methods.

In Table 7, we show the case *p* = 2 (Gaussianity) with heteroskedastic errors, where we estimated the same models as before ARMA (1,1), GARCH (1,1), t-GARCH (1,1) and GED-GARCH (1,1).

In that case the best model is the GARCH (1,1) because it has the lowest MSFE for phi = 0.2; phi = 0.4 and for phi = 0.6. It is well known that when the shape parameter *p*, is equal to 2, the GED distribution is equivalent to the Gaussian distribution. The most accurate forecast combination method is the Lp-norm for the cases phi = 0.6 and phi = 0.8 (that means phi medium-large). On the contrary when we have phi = 0.2 and phi = 0.4 (medium–low values of phi) the best forecast combination is the OLS.

From Table 8 that illustrates the case p = 2.5 (leptokurtic case) we can observe that the best forecasting model is the GARCH (1,1) for three cases on four (phi = 0.2 and phi = 0.4 and phi = 0.6).

For the case phi = 0.8 the best forecast model is the ARMA (1,1). In two cases of the four we considered the Lp-norm provides the most accurate forecast combination. In particular the good performance of the Lp method occurs for the central values of the phi parameter (phi = 0.4 and phi = 0.6). Indeed the OLS method furnish us the best forecasting combination for extreme values of phi parameter (Phi = 0.2; Phi = 0.8) Hence, we can conclude that in general in the case of homoskedastic innovations, the Lp-norm method always provides the most accurate forecasts considering the different of values p (1.5; 2; 2.5) and different values of Phi (0.2; 0.4; 0.6; 0.8). Completely different is the situation with heteroskedastic innovation. In this cases Lp-norm is better than CLS and LAD, but it is not always better than the OLS, indeed sometimes the last one performs better than Lp-norm method. Moreover the difference in accuracy between Lp-norm and OLS is really small so we might not care.

## Real data application

For real data application, we analyzed 26 time series. One of these was examined individually, while we used the other 25 to reinforce and generalize what we observed from the study of a single time series.

### Historical series of Bitcoin

Cerqueti et al. [11] recently propose a new and comprehensive study about cryptocurrency market. At this aim, they have considered non-Gaussian GARCH volatility models, which formed a class of stochastic recursive systems commonly adopted for financial predictions. Results have shown that the best specification and forecasting accuracy are achieved under the Skewed Generalized Error Distribution when Bitcoin/USD and Litecoin/USD exchange rates are considered, while the best performances are obtained for Skewed Distribution in the case of Ethereum/USD exchange rate.

Taking this research as a reference point, we used an historical daily financial series of the BTC/USD exchange rate from Bitfinex ranging from March 1st, 2014 to February 28th, 2020. Bitfinex is one of the best cryptocurrency trading websites.

Bitcoin is not classified as a currency, but rather as an highly variable way of transacting because its value is not defined by a central bank but only by supply and demand (1 bitcoin was worth 300 EUR in 2012, it peaked at 14,000 EUR in 2018 and by the time of writing it was worth 10,000 EUR).

We took that historical series from investing.com, a financial portal that tracks down the financial performance of companies around the world. One of the most recent and interesting research about the potential to improve accuracy with forecast combinations and its real applications has been given by Liu et al. [35] and Shaub [44].

The volatility models for cryptocurrencies have not been fully developed yet, and, most of all, the previous literature on this subject [8] has been focused on finding the best GARCH model ignoring the assumption about the error distribution, even if the characteristics of non-normality and asymmetry of the distribution of Bitcoin's return are well known to researchers [8]. Our aim, with this application, is to verify that the combination of forecasts generates more accurate results (specifically, we intend to prove that the Lp-norm combination method is better than the others in situations of multicollinearity and in presence of non-Gaussian distribution) to verify the good performance of the GED distribution. To reach these targets we used the software R.

**Fig. 2:**
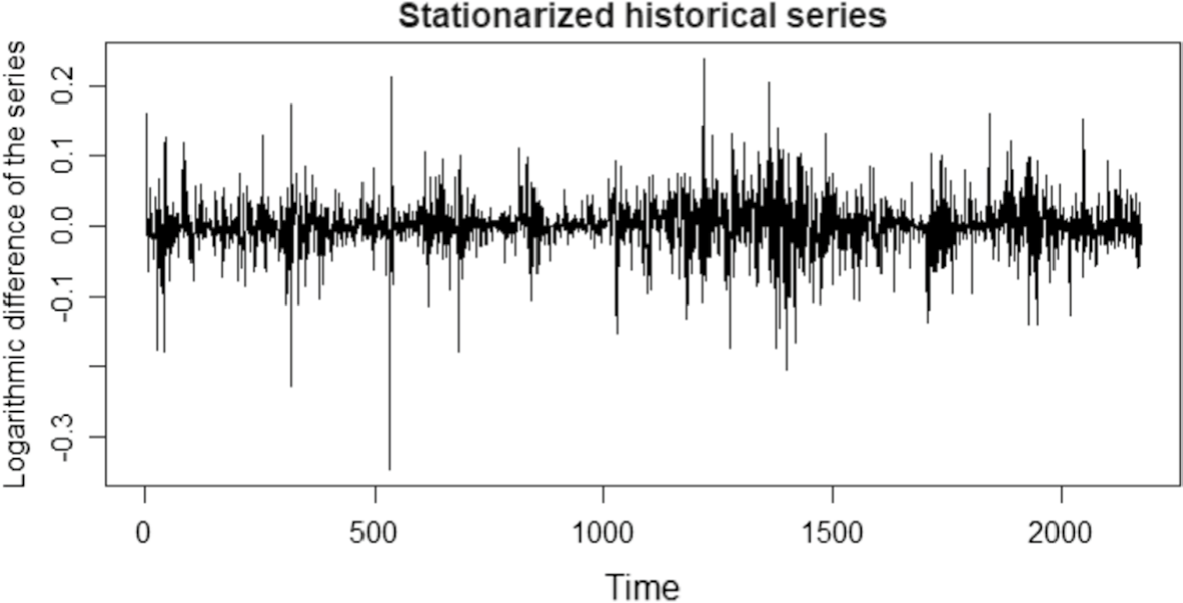
Stationary logarithmic historical series

We start our application showing the descriptive statistics (Table 9) and the stationary logarithmic historical series (Fig. 2).

**Table 9:** Descriptive statistics of Historical series of Bitcoin

Variables	Mean	Min	Max	Median	Std. Dev
Historical Series	3623.4	183	19,187	1110.6	3915.598
Log. Historical Series	7.365	5.210	9.862	7.013	1.408027
First Difference Log. Historical Series	0.001256	− 0.345316	0.237220	0.001096	0.03952906

Source: own elaboration

Firstly, we verified that the series have been stationary with the Dickey–Fuller test and the KPSS test and, of course, the historical series of returns are always stationary (Fig. 2).

Then, after splitting the historical series in the training set (or 80% of distribution data) and testing set (20% of distribution data) and we estimated the best ARIMA model for the training set that is the ARIMA (2,0,0) with coefficients AR1 = − 0.0302 and AR2 = − 0.0139.

Successively, we fitted the training set with three GARCH (1,1): one with Gaussian distribution (with coefficients of the fitted model: AR1 = − 0.45 and MA1 = 0.526), one with t Student distribution (with coefficients: AR1 = 0.342 and MA1 = − 0.413) and one with GED distribution (with coefficients: AR1 = 0.131 and MA1 = − 0.23). So, we estimated the forecasts from four separate data generating processes. We estimated 543 forecasts (the testing set) of the model fitted on the training set of the distribution and then we compared them through the MSFE between the forecasts and the elements of the testing set.

The best forecast is given by the GED GARCH (1,1) because it has the smallest MSFE. So we have proved that, among the individual models, the best is the GED GARCH (1,1) (Table 10).

**Table 10:** Root Mean Square Error for different models obtained from an historical daily financial series of the BTC/USD exchange rate

AR(2)	Gaussian GARCH (1,1)	t-student GARCH (1,1)	GED GARCH (1,1)
0.03541969	0.03540635	0.03540162	**0.03539828**

Source: own elaboration

Smaller value of Root Mean Square Error between different models in bold

We did the Jarque Bera test on the residuals of GED-GARCH (1,1) and the result was that the distribution of the residuals of GED-GARCH (1,1) is not Gaussian because the p value is the very low (2.2e−16).

As we can see from the QQ plots and from the results of the Jarque Bera test (that consider the null hypothesis H_0_ = Gaussianity), our sample is not Gaussian. This is why, among the individual models, the GED GARCH (1,1) is the best in order to make forecasts.

The last thing we needed to prove was that the Lp norm is the best combination method in situations in situations like the ones of multicollinearity and non-Gaussian distribution. We combined the four models with OLS, CLS, LAD and an Lp-norm combination. By doing that, we confirmed that the MSFE was smaller than the MSFE for the individual models (Table 11).

**Table 11:** Root Mean Square Error for different methods of combination model obtained from an historical daily financial series of the BTC/USD exchange rate

OLS combination	CLS combination	LAD combination	Lp-norm combination
0.03513259	0.03539686	0.03513391	**0.03513258**

Source: own elaboration

Smaller value of Root Mean Square Error between different models in bold

As we can see, every MSFE is smaller than the MSFE of the individual models so we can assert that the combination method of forecasts generates more accurate results.

**Fig. 3:**
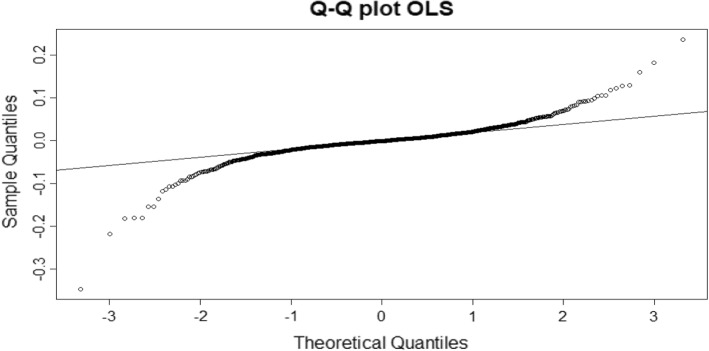
QQ-plot of GARCH (1,1) with a GED distribution. Source: own elaboration

**Fig. 4:**
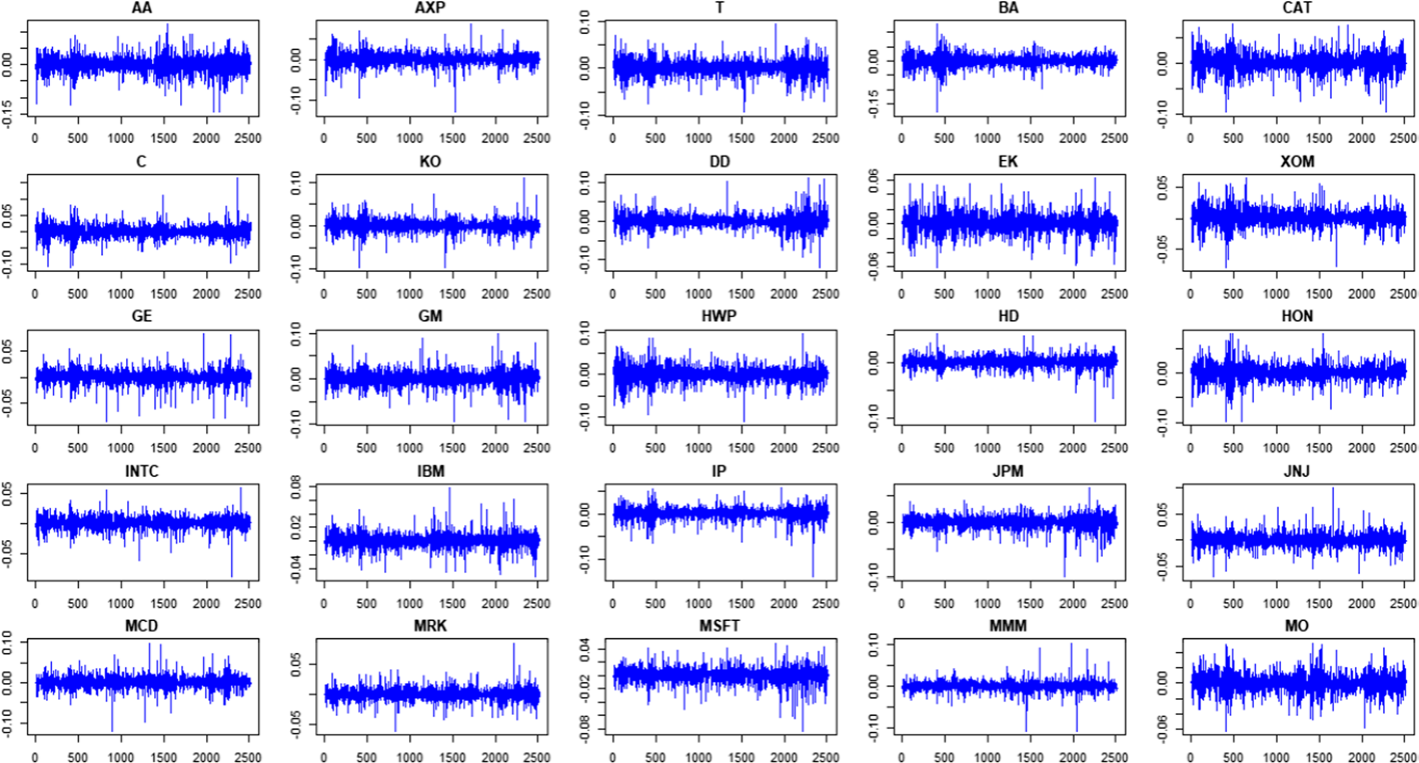
Stationary logarithmic historical series. Source: own elaboration

The best combination method for the forecastings, in that case, are either the OLS or the Lp-norm. They only differ for $1\times {10}^{-8}$, so we can maintain that these two forecast combination models are equal. As proof of this we can see the complete similarity of Figs. 3 and 4 which represent the two analyzed Q–Q plots. For this reason we assume that Lp-norm method can be considered more general than OLS (giving approximately the some results) and we decided to compare the last one with CLS and LAD considering 25 further hystorical series.

### Application to Dow Jones index

To verify the predictive quality of the proposed method, we considered 25 historical series (taken from Yahoo Finance) listed in the Dow Jonson index, which measures the performance of 30 companies representing about a quarter of the value of the securities traded on the New York Stock Exchange (NYSE). We excluded from our study the series with missing values, so we used 25 historical series of 2516 observations (from 2010/01/01 to 2020/01/01).

Moreover we could considered more observations, but we have chosen to stop the historical series in the first day of 2020 because of the Covid-19 global pandemic that caused a lot of problems in the market stability, otherwise the forecasts would have been meaningless.

**Fig. 5:**
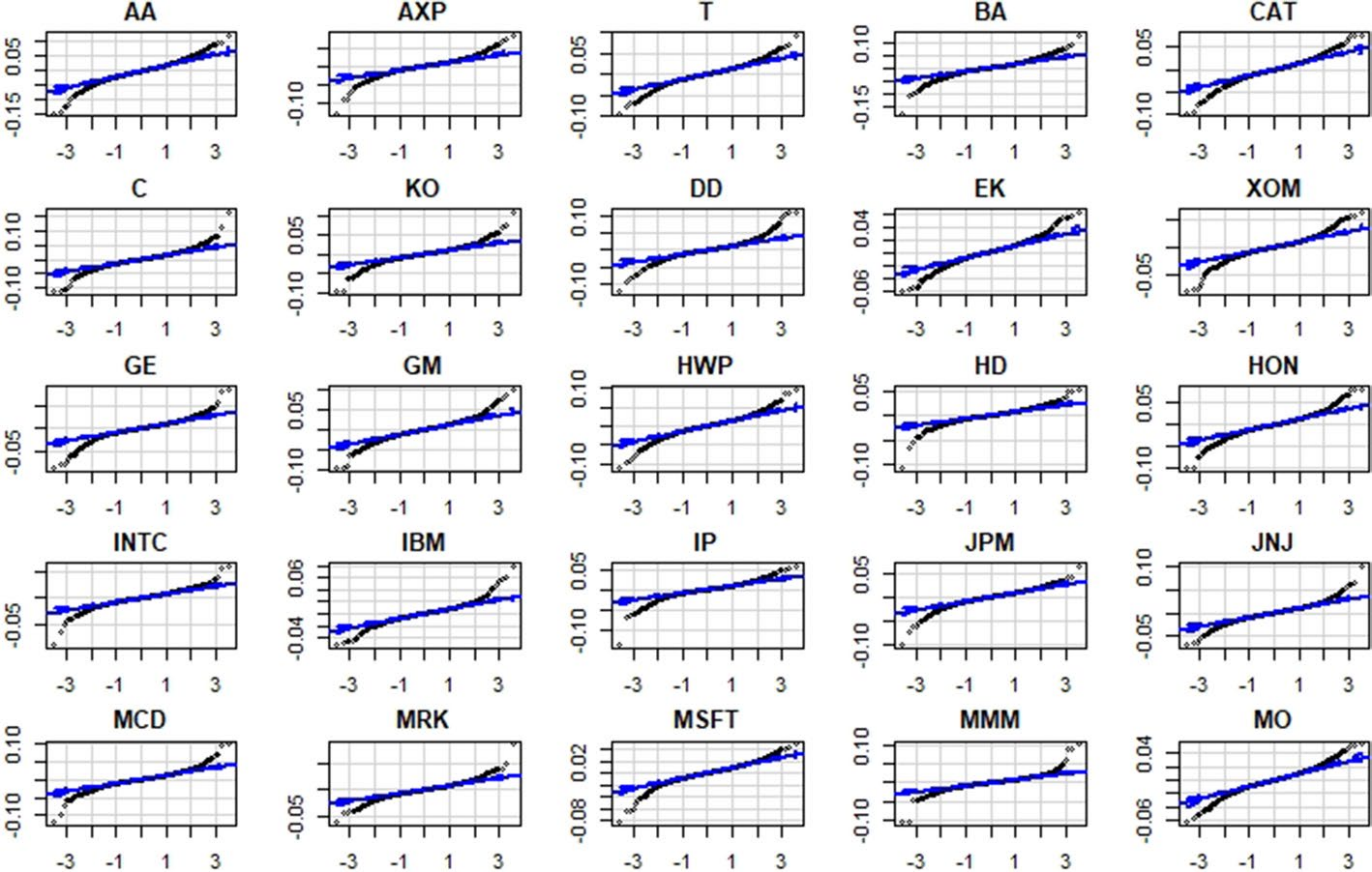
Q–Q plots of time series returns. Source: own elaboration

Below in Fig. 5 we show the historical time series with logarithmic standardization and the Q-Q plots of time series returns:

Looking at the Q-Q plots we can see that the behavior of the series should be very close to Gaussian distribution. In fact the curve are very close to the Gaussianity lines and consequently our distributions (at least in the 25 cases considered) seems similar to the normal distributions.

In the following table (Table 12) are reported the descriptive statistics.

**Table 12:** Descriptive statistics of the 25 historical series, taken from Yahoo Finance listed in the Dow Jonson index

	Min	1Q	Median	Mean	3Q	Max
AA	− 0.14519	− 0.01273	0	− 0.00022	0.012858	0.124116
AXP	− 0.12898	− 0.00598	0.000758	0.000502	0.008035	0.086445
BA	− 0.09353	− 0.00738	0.001076	0.000795	0.009513	0.094214
C	− 0.17934	− 0.0087	0.000387	0.000371	0.009744	0.129678
CAT	− 0.09674	− 0.00785	0.000402	0.000477	0.009637	0.077955
DD	− 0.11008	− 0.0089	0.000449	0.00029	0.009852	0.163949
DIS	− 0.09619	− 0.0058	0.000779	0.000654	0.007544	0.109247
GE	− 0.11986	− 0.00769	0	366E+08	0.007796	0.110184
HD	− 0.06069	− 0.0052	0.000919	0.000901	0.007454	0.062148
HON	− 0.08002	− 0.00548	0.000682	0.000696	0.007097	0.064608
IBM	− 0.08642	− 0.00581	0.000381	0.000122	0.006479	0.084934
INTC	− 0.09543	− 0.00775	0.000678	0.000541	0.008868	0.100315
IP	− 0.11224	− 0.00866	0.000568	0.000349	0.010256	0.097621
JNJ	− 0.10578	− 0.00393	0.000366	0.000443	0.005642	0.052422
JPM	− 0.09888	− 0.00716	0.000521	0.000564	0.008682	0.081012
KO	− 0.08813	− 0.00441	0.000484	0.000386	0.005524	0.058947
MCD	− 0.05173	− 0.00452	0.00083	0.000574	0.005722	0.078105
MMM	− 0.13863	− 0.00486	0.000707	0.000402	0.006727	0.057447
MO	− 0.09973	− 0.00499	0.001102	0.000568	0.006973	0.064465
MRK	− 0.06851	− 0.00585	0.000476	0.000496	0.007294	0.099013
MSFT	− 0.12103	− 0.00663	0.000589	0.000742	0.008179	0.099413
PG	− 0.0606	− 0.00428	0.000373	0.000409	0.005351	0.084328
T	− 0.08399	− 0.00514	0.000847	0.000342	0.006361	0.048764
WMT	− 0.1074	− 0.00491	0.000669	0.00041	0.005991	0.103444
XOM	− 0.06388	− 0.00599	0.000113	0.000132	0.006733	0.053692

Source: own elaboration

In what follows we provide the result related to the forecast accuracy of the different regression-based combination approaches.

How we can see in Table 13, the ARMA (1,1) is the best model for most of the historical because it has the smaller MSFE in 15 cases. In the other historical series seven times the GARCH (1,1) is the best model and only three times the GED-GARCH (1,1) is the best one.

It is known that, combining forecasts increases the accuracy, indeed if we compare Table 13 with Table 14, we notice that the MSFE of the single forecasts are always greater than the MSFE for the combined forecasts.

**Table 13:** Root Mean Square Error for single forecasts of different models taken from the 25 tickers of NISE securities listed in the Dow Jonson index

	ARMA (1,1)	GARCH (1,1)	t-GARCH (1,1)	GED-GARCH (1,1)
AA	0.026843271	0.026842777	0.026842071	**0.0268381**
AXP	**0.013176035**	0.013185607	0.013180915	0.013179585
BA	**0.019016242**	0.019025106	0.019027594	0.019028431
C	**0.015316327**	0.015323952	0.015321853	0.015318666
CAT	**0.018906329**	0.018916453	0.018916454	0.018909249
DD	**0.019449013**	0.019481562	0.019479437	0.019473862
DIS	**0.013608562**	0.013608688	0.013610309	0.013609062
GE	**0.025351524**	0.02535264	0.025352249	0.025352486
HD	0.012963545	0.01297067	0.012967218	**0.012962132**
HON	**0.011583486**	0.011593648	0.011587492	0.011586781
IBM	**0.01434896**	0.014349976	0.014352857	0.014351017
INTC	0.019309015	**0.019308887**	0.019309435	0.01930921
IP	**0.016904549**	0.016910866	0.016915275	0.016907335
JNJ	0.012331075	0.012336865	0.012333638	**0.012330256**
JPM	**0.012913236**	0.012922966	0.012918259	0.012916045
KO	0.010132191	**0.010131339**	0.010131361	0.010131353
MCD	0.01137472	**0.011374755**	0.01137923	0.011382468
MMM	**0.01562664**	0.015633654	0.015651649	0.015637037
MO	**0.015406586**	0.015416791	0.01543804	0.015435169
MRK	0.012079222	**0.012065029**	0.012072872	0.01207854
MSFT	0.015354279	**0.015350535**	0.015354094	0.015361863
PG	0.011292441	**0.011288397**	0.01128993	0.011291204
T	0.013301173	**0.013300923**	0.013304403	0.013305561
WMT	**0.012464111**	0.012465047	0.012464727	0.012464904
XOM	**0.012734256**	0.01274105	0.012738435	0.012735775

Bold font highlights the models with the lowest MSFE for each single

**Table 14:** Root Mean Square Error for different methods of forecast combination for the 25 tickers of NISE securities listed in the Dow Jonson index

	CLS	LAD	Lp-norm
AA	0.026838	0.026758	**0.026742**
AXP	0.013176	0.013157	**0.013156**
BA	0.019016	0.018919	**0.018917**
C	0.015316	0.015312	**0.015307**
CAT	0.018906	0.018864	**0.018861**
DD	0.019449	0.019374	**0.019363**
DIS	0.013609	0.01351	**0.013502**
GE	0.025352	0.025279	**0.025277**
HD	0.012962	0.01295	**0.012943**
HON	0.011583	0.011569	**0.011562**
IBM	0.014349	0.014276	**0.014268**
INTC	0.019309	0.01923	**0.019219**
IP	0.016905	0.016816	**0.016806**
JNJ	0.01233	0.012329	**0.012322**
JPM	0.012913	0.012892	**0.012891**
KO	0.010131	0.010108	**0.010107**
MCD	0.011375	0.011383	**0.011366**
MMM	0.015627	0.015618	**0.01559**
MO	0.015407	0.015357	**0.015348**
MRK	0.012065	0.012053	**0.01204**
MSFT	0.015351	0.015346	**0.01533**
PG	0.011288	0.011272	**0.011266**
T	0.013301	0.013257	**0.013249**
WMT	0.012464	0.012467	**0.012457**
XOM	0.012734	0.012682	**0.012679**

Bold font highlights the models with the lowest MSFE for each forecast combination scheme

The combination of the forecasts led to very satisfactory results, we investigated the potential of the proposed methodology on a large segment of the NYSA, obtaining forecasts with an MSFE (Table 14) smaller than both of the compared methodologies. In fact, the Lp-norm estimators make always better predictions than CLS and LAD. The Lp-norm provides the most accurate results for all of the considered stock-returns-data.

## Concluding remarks

This paper started with the aim of evaluating whether the method of combining forecasts with the Lp-norm constituted a valid alternative to the most common existing methods.

Through the analysis of the simulations, we found that the combination of forecasts with Lp-norm generates an MSFE lower than the OLS, LAD and CLS methods in situations of strong multicollinearity and non-Gaussianity especially in presence of homo-skedastics innovations.

In this case the Lp-norm provides the most accurate forecast with respect to the alternative regression-based combination methods.

In the case of eteroskedastic innovations instead, the Lp-norm performs better for highly persistent process (phi = 0.8) or for poorly persistent process (phi = 0.2) regarding the in leptokurtic data (p = 1.5) (see Table 12).

In the case of Gaussian data (*p* = *2*) the Lp-norm seems to perform better for a highly level of persistency (phi = 0.6 and phi = 0.8) (Table 13). Finally concerning the platikurtic data (*p* = *2.5*) the Lp-norm provides the best forecast combination for a medium level of persistency (phi = 0.4 and phi = 0.6) (Table 14).

On the other hand, by analysing the empirical data, the MSFE using the combination with Lp-norm confirmed the result of the simulation: it was lower than the one obtained with the LAD and CLS methods, while, comparing it to the MSFE of the combination with the OLS (historical series of Bitcoin), we obtained an almost infinitesimal difference (Table 15).

**Table 15:** Equity securities classification captions

**AAL** Anglo American PLC	**ADM** Admiral Group	**AHT** Ashtead Group	**AUTO** Auto Trader Group	**AZN** Astra Zeneca PLC
**BHP** BHP Group	**BME** B&M	**CPG** Compass Group	**CRH** CRH PLC	**DGE** Diageo PLC
**EVR** Evzar PLC	**FERG** Ferguson PLC	**GSK** Glaxosmithkline PLC	**IAG** Internetional Airlines Group	**IHG** IHG Hotels & Resorts PLC
**III** 3I Group PLC	**MNG** M&G PLC	**MRO** Melrose Industries PLC	**NWG** Natwest Group PLC	**PRU** Prudential PLC
**RIO** Rio Tinto Group	**SVT** Severn Trent PLC	**TSCO** Tesco PLC	**VOD** Vodafone Group	**WPP** Wire and Plastic Products PLC

Source: https://www.londonstockexchange.com/indices/ftse-100/constituents/table

The reason of this small difference is due to the sample size since due to the central limit theorem there is a tendency to Gaussianity, which is compliant with the results from simulation, that is to say in the presence of a normal distribution. Looking at the 25 historical series relating to companies included in the Dow Jonson we can observe a strong tendency to Gaussianity (confirmed by the QQ-Plot analysis) that means the same behaviour of OLS and Lp-norm forecast combinations.

In conclusion, the method assuming Lp-norm estimators is a valid method of combining forecasts since, compared to other ones (LAD and CLS), it generates a lower MSFE.

However, in the Gaussian situation, it can constitute an alternative to the OLS while providing excellent results in terms of MSFE as the difference is minimal.

## Figures and Tables

**Fig. 6:**
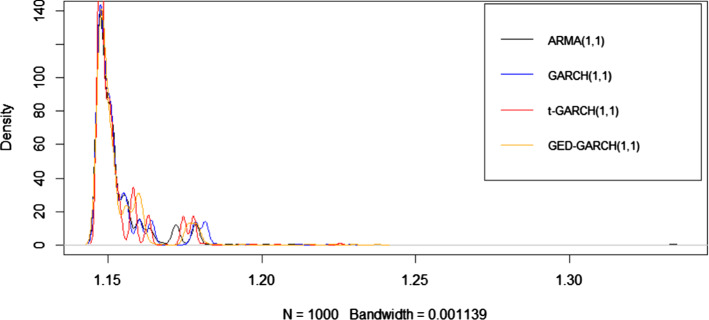
Time series with 1000 simulated paths, homoskedastic errors, length 200, autoregressive process 0.2, p = 1.5, Root Mean Square Error: densities. Source: own elaboration

**Fig. 7:**
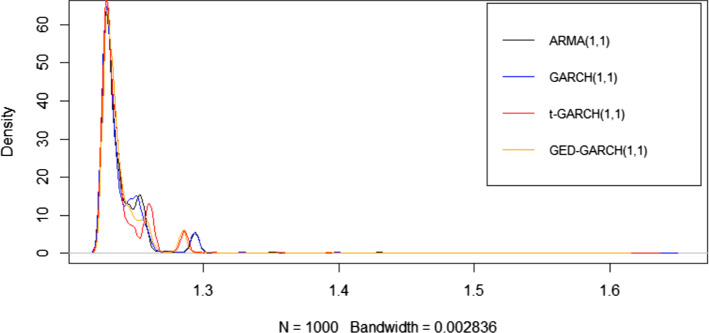
Time series with 1000 simulated paths, homoskedastic errors, length 200, autoregressive process 0.4, p = 1.5, Root Mean Square Error: densities. Source: own elaboration

**Fig. 8:**
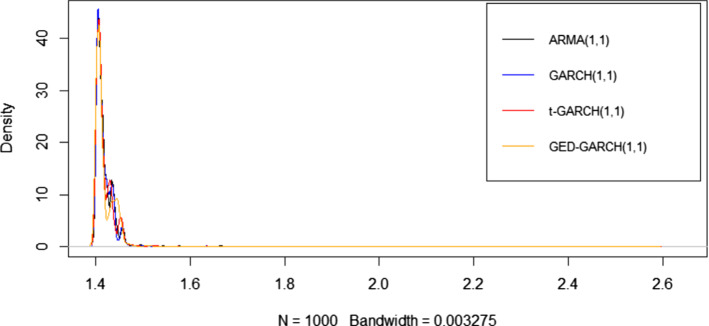
Time series with 1000 simulated paths, homoskedastic errors, length 200, autoregressive process 0.6, p = 1.5, Root Mean Square Error: densities. Source: own elaboration

**Fig. 9:**
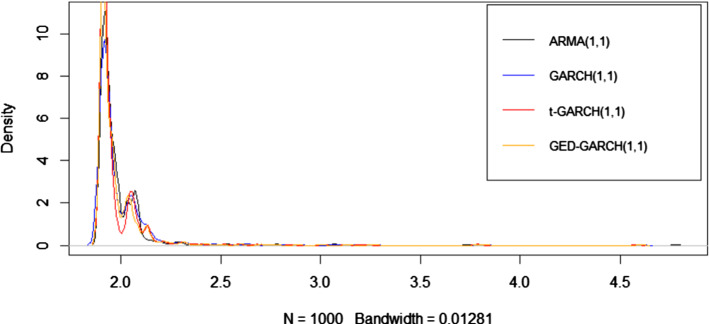
Time series with 1000 simulated paths, homoskedastic errors, length 200, autoregressive process 0.8, p = 1.5, Root Mean Square Error: densities. Source: own elaboration

**Fig. 10:**
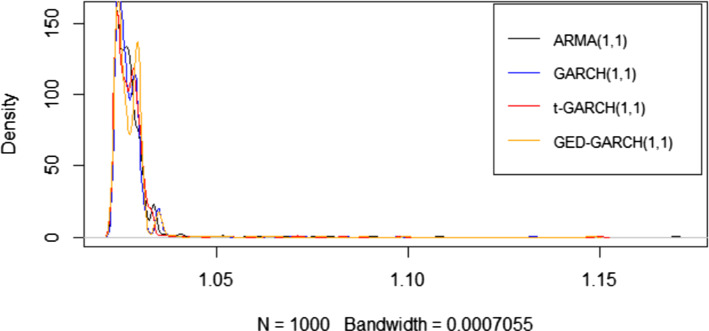
Time series with 1000 simulated paths, homoskedastic errors, length 200, autoregressive process 0.2, p = 2, Root Mean Square Error: densities. Source: own elaboration

**Fig. 11:**
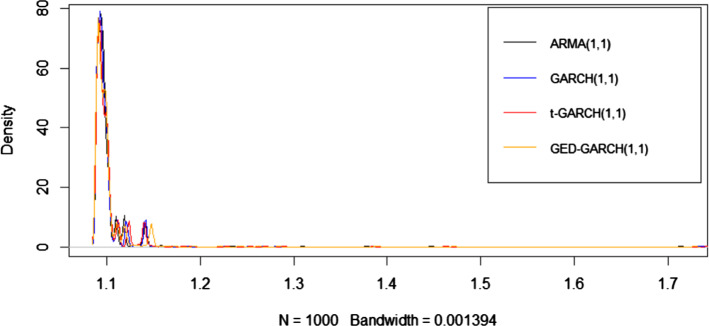
Time series with 1000 simulated paths, homoskedastic errors, length 200, autoregressive process 0.4, p = 2, Root Mean Square Error: densities. Source: own elaboration

**Fig. 12:**
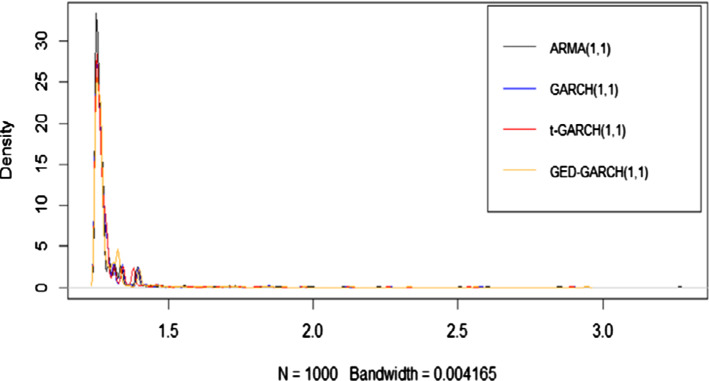
Time series with 1000 simulated paths, homoskedastic errors, length 200, autoregressive process 0.6, p = 2, Root Mean Square Error: densities. Source: own elaboration

**Fig. 13:**
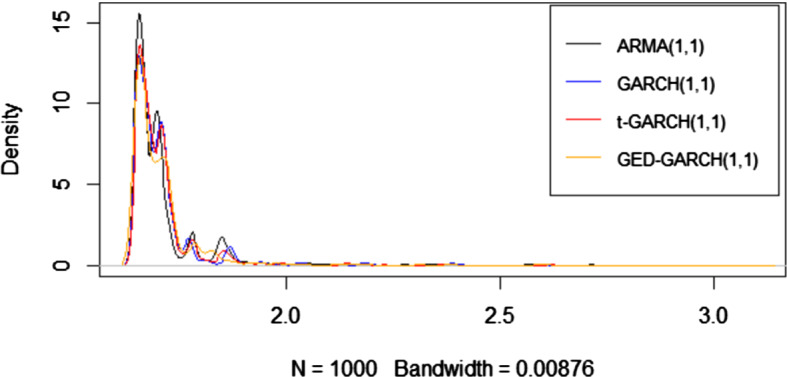
Time series with 1000 simulated paths, homoskedastic errors, length 200, autoregressive process 0.8, p = 2, Root Mean Square Error: densities. Source: own elaboration

**Fig. 14:**
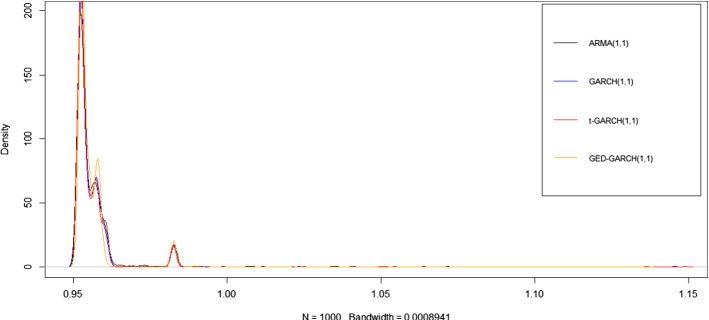
Time series with 1000 simulated paths, homoskedastic errors, length 200, autoregressive process 0.2, p = 2.5, Root Mean Square Error: densities. Source: own elaboration

**Fig. 15:**
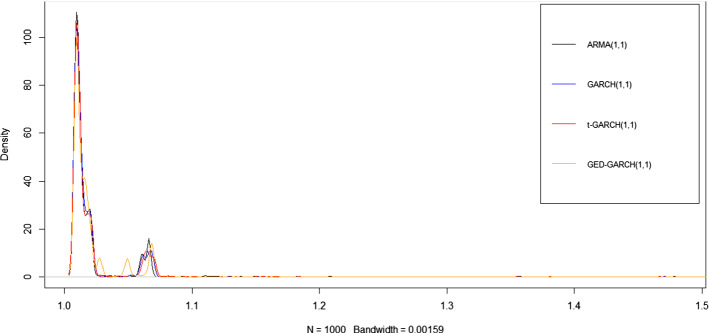
Time series with 1000 simulated paths, homoskedastic errors, length 200, autoregressive process 0.4, p = 2.5, Root Mean Square Error: densities. Source: own elaboration

**Fig. 16:**
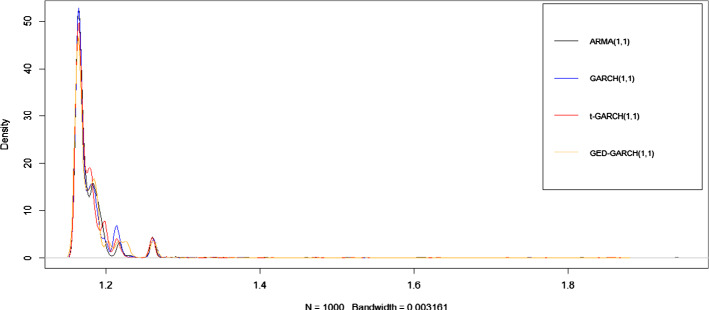
Time series with 1000 simulated paths, homoskedastic errors, length 200, autoregressive process 0.6, p = 2.5, Root Mean Square Error: densities. Source: own elaboration

**Fig. 17:**
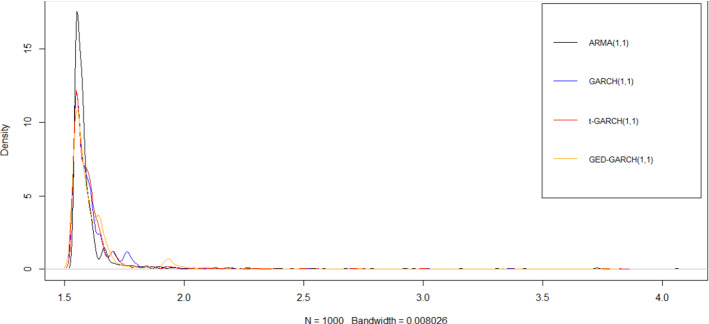
Time series with 1000 simulated paths, homoskedastic errors, length 200, autoregressive process 0.8, p = 2.5, Root Mean Square Error: densities. Source: own elaboration

**Fig. 18:**
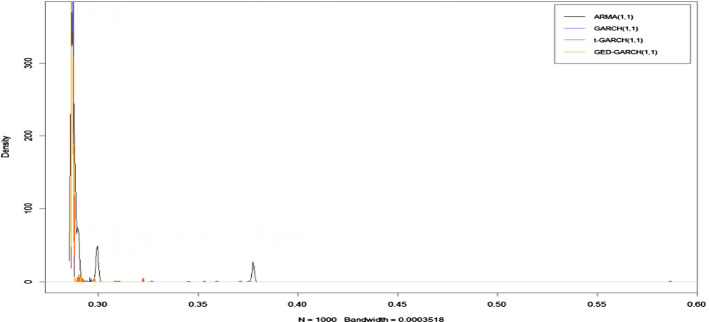
Time series with 1000 simulated paths, heteroskedastic errors, length 200, autoregressive process 0.2, p = 1.5, Root Mean Square Error: densities. Source: own elaboration

**Fig. 19:**
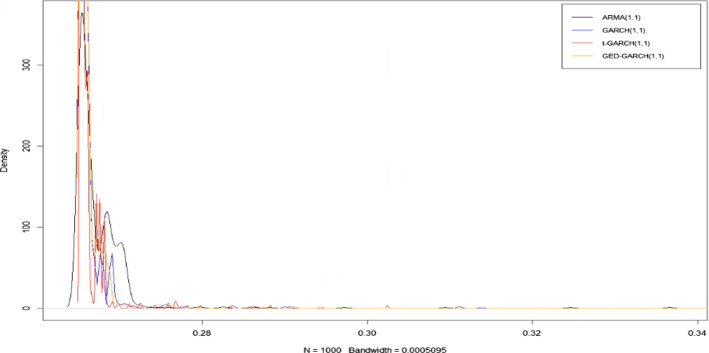
Time series with 1000 simulated paths, heteroskedastic errors, length 200, autoregressive process 0.4, p = 1.5, Root Mean Square Error: densities. Source: own elaboration

**Fig. 20:**
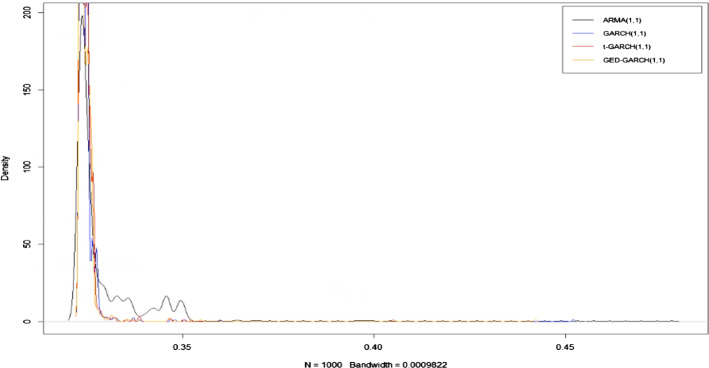
Time series with 1000 simulated paths, heteroskedastic errors, length 200, autoregressive process 0.6, p = 1.5, Root Mean Square Error: densities. Source: own elaboration

**Fig. 21:**
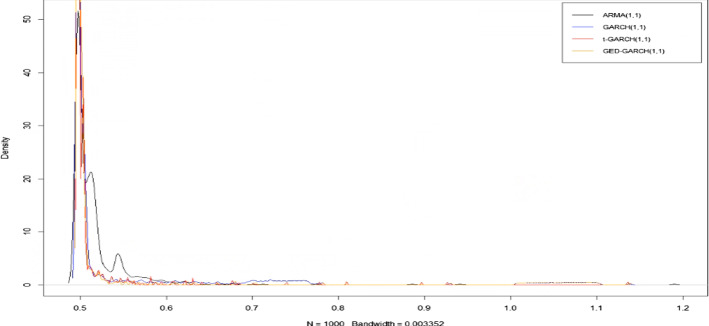
Time series with 1000 simulated paths, heteroskedastic errors, length 200, autoregressive process 0.8, p = 1.5, Root Mean Square Error: densities. Source: own elaboration

**Fig. 22:**
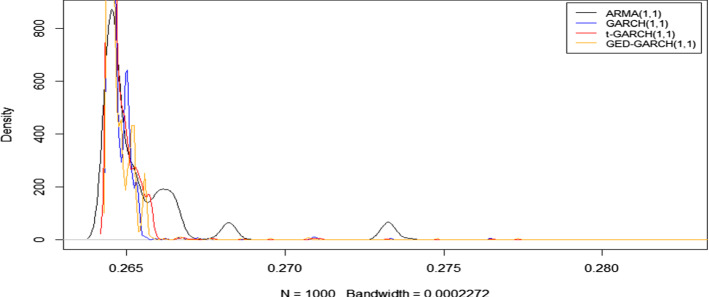
Time series with 1000 simulated paths, heteroskedastic errors, length 200, autoregressive process 0.2, p = 2, Root Mean Square Error: densities. Source: own elaboration

**Fig. 23:**
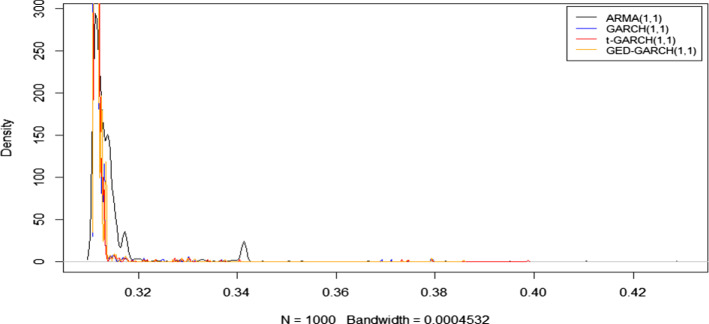
Time series with 1000 simulated paths, heteroskedastic errors, length 200, autoregressive process 0.4, p = 2, Root Mean Square Error: densities. Source: own elaboration

**Fig. 24:**
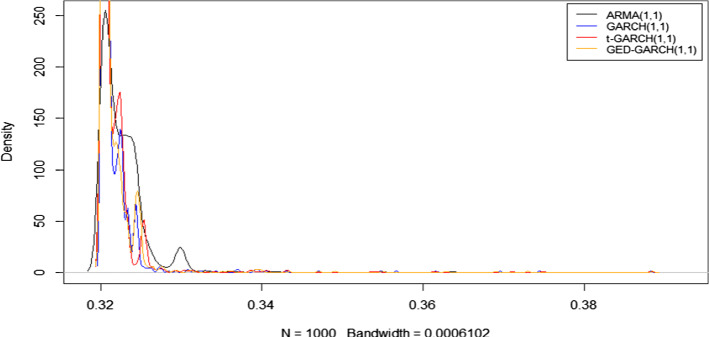
Time series with 1000 simulated paths, heteroskedastic errors, length 200, autoregressive process 0.6, p = 2, Root Mean Square Error: densities. Source: own elaboration

**Fig. 25:**
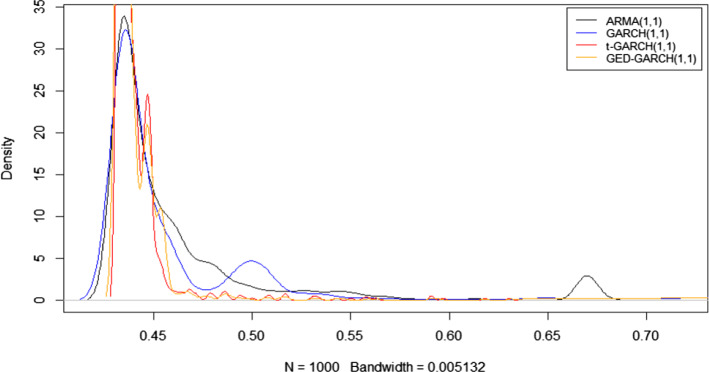
Time series with 1000 simulated paths, heteroskedastic errors, length 200, autoregressive process 0.8, p = 2, Root Mean Square Error: densities. Source: own elaboration

**Fig. 26:**
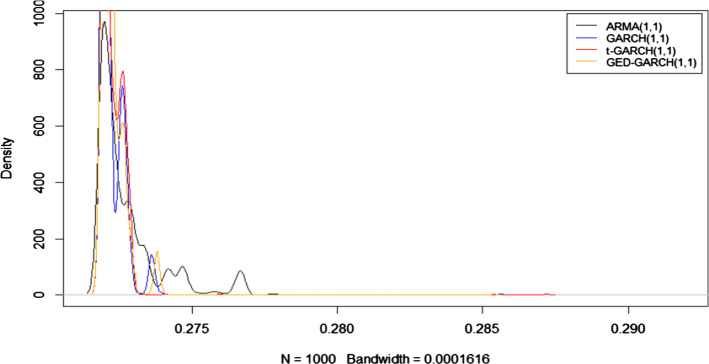
Time series with 1000 simulated paths, heteroskedastic errors, length 200, autoregressive process 0.2, p = 2.5, Root Mean Square Error: densities. Source: own elaboration

**Fig. 27:**
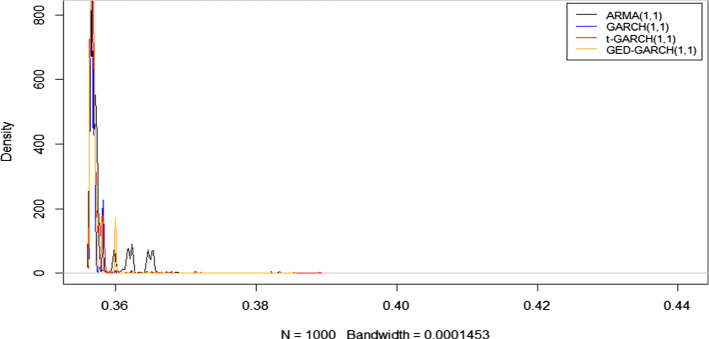
Time series with 1000 simulated paths, heteroskedastic errors, length 200, autoregressive process 0.4, p = 2.5, Root Mean Square Error: densities. Source: own elaboration

**Fig. 28:**
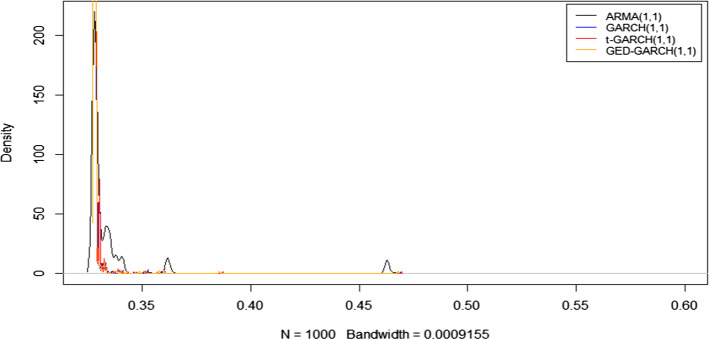
Time series with 1000 simulated paths, heteroskedastic errors, length 200, autoregressive process 0.6, p = 2.5, Root Mean Square Error: densities. Source: own elaboration

**Fig. 29:**
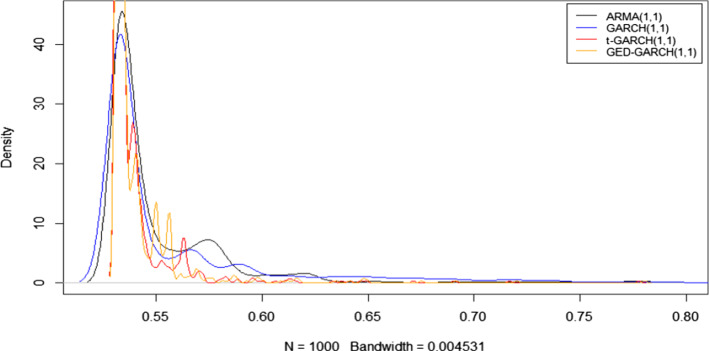
Time series with 1000 simulated paths, heteroskedastic errors, length 200, autoregressive process 0.8, p = 2.5, Root Mean Square Error: densities. Source: own elaboration

## Appendix 1

**R procedure used for simulations:**

**Load necessary packages, fit AR(1) like DGP with a GED distribution of the innovation, split the AR(1) fitted in training and testing set, fit an ARMA(1,1) on the training set, heteroscedasticity test. **

library(tseries)

library(timeSeries)

library(forecast)

library(normalp)

library(TSA)

library(rugarch)

library(ForecastComb)

library(rio)

library(BatchGetSymbols)

library(xtable)

library(foreach)

library(doParallel)

**We use parallel computation for the simulations.**

cl<-makeCluster(detectCores()-2)

registerDoParallel(cl)

**Setting the seed for results' replication.**

set.seed(1000)

t<-200 length of the simulated time series (default T=200)

p<-1.5 shape parameter of the GED distribution (default p=1.5)

phi<-0.2 autoregressive (persistence) parameter (default phi=0.2)

f<-0.8 training set length (default=80%)

**For an AR(1) process with homoskedastic errors:**

sim_data<-matrix(NA, nrow=t, ncol=1000)

for (i in 1:1000){

sim_data[,i]<-arima.sim(n=t,model=list(order=c(1,0,0),ar=phi), innov=rnormp(1000,0,1,p))

}

colnames(sim_data)<-as.character(seq(1:1000))

**Instead, for an AR(1) process with heteroskedastic errors:**

sim_data<-matrix(NA, nrow=t, ncol=1000)

for (i in 1:1000){

sim_data[,i]<-arima.sim(n=200,model=list(order=c(1,0,0),ar=phi), innov=garch.sim(alpha=c(0.01,0.9), n = 1000, rnd = rnorm))

}

colnames(sim_data)<-as.character(seq(1:1000))

**Splitting training and testing:**

data_train<-sim_data[1:(t*f),]

data_test<-sim_data[(t*f+1):t,]

**Fit the training set with three GARCH(1,1) with normal, T-student and GED distribution of innovation.**

spec1<-ugarchspec(variance.model = list(model = "sGARCH",garchOrder = c(1, 1)), mean.model = list(armaOrder = c(1, 0)), distribution.model = "norm")

spec2<-ugarchspec(variance.model = list(model = "sGARCH", garchOrder = c(1, 1)), mean.model = list(armaOrder = c(1, 0)),distribution.model = "std")

spec3<-ugarchspec(variance.model = list(model = "sGARCH", garchOrder = c(1, 1)), mean.model = list(armaOrder = c(1, 0)),distribution.model = "ged")

**Forecast with ARMA(1,1), GARCH(1,1) with normal, T-student and GED distribution of innovation.**

ARMA<-matrix(NA, nrow=(1-f)*t+1, ncol=1000)

garchN<-matrix(NA, nrow=(1-f)*t+1, ncol=1000)

garchT<-matrix(NA, nrow=(1-f)*t+1, ncol=1000)

garchG<-matrix(NA, nrow=(1-f)*t+1, ncol=1000)

ARMA<-foreach(i = 1:1000, .combine = cbind, .packages= "forecast") %dopar% {

preds<-forecast(Arima(data_train[,i], order=c(1,0,1)), h=(1-f)*t+1)$mean

preds

}

garchN<-foreach(i = 1:1000, .combine = cbind, .packages= "rugarch") %dopar% {

preds<-ugarchforecast(ugarchfit(spec1, data_train[,i], solver = 'hybrid'), n.ahead = 40)@forecast$seriesFor

preds

}

garchT<-foreach(i = 1:1000, .combine = cbind, .packages= "rugarch") %dopar%

{

preds<-ugarchforecast(ugarchfit(spec2, data_train[,i], solver = 'hybrid'), n.ahead = 40)@forecast$seriesFor

preds

}

garchG<-foreach(i = 1:1000, .combine = cbind, .packages= "rugarch") %dopar% {

preds<-ugarchforecast(ugarchfit(spec3, data_train[,i], solver = 'hybrid'), n.ahead = 40)@forecast$seriesFor

preds

}

**MSFE of the individual forecasts.**

MSFE1<-vector(mode="numeric", length=1000)

MSFE2<-vector(mode="numeric", length=1000)

MSFE3<-vector(mode="numeric", length=1000)

MSFE4<-vector(mode="numeric", length=1000)

for (i in 1:1000) {

MSFE1[i]<-sqrt(mean((ARMA[i]-data_test)^2))

MSFE2[i]<-sqrt(mean((garchN[i]-data_test)^2))

MSFE3[i]<-sqrt(mean((garchT[i]-data_test)^2))

MSFE4[i]<-sqrt(mean((garchG[i]-data_test)^2))

}

plot(density(MSFE1), main="Root Mean Square Error: densities", type="l")

lines(density(MSFE2), col="blue")

lines(density(MSFE3), col="red")

lines(density(MSFE4), col="orange")

legend("topright", legend=c("ARMA(1,1)", "GARCH(1,1)", "t-GARCH(1,1)", "GED-GARCH(1,1)"),col=c("black", "blue", "red", "orange"), lty=1:1, inset=0.01, cex=0.8)

mean(MSFE1) # MSFE of the ARMA(1,1)

mean(MSFE2) # MSFE of the Gaussian-GARCH(1,1)

mean(MSFE3) # MSFE of the t-GARCH(1,1)

mean(MSFE4) # MSFE of the GED-GARCH(1,1)

**Forecast combination.**

results<-matrix(NA, nrow = 1000, ncol=4)

for (i in 1:1000) try({

results[i,1]<-comb_OLS(foreccomb(data_test[,i], cbind(ARMA[,i], garchN[,i], garchT[,i], garchG[,i])))$Accuracy_Train[2]

results[i,2]<-comb_CLS(foreccomb(data_test[,i], cbind(ARMA[,i], garchN[,i], garchT[,i], garchG[,i])))$Accuracy_Train[2]

results[i,3]<-comb_LAD(foreccomb(data_test[,i], cbind(ARMA[,i], garchN[,i], garchT[,i], garchG[,i])))$Accuracy_Train[2]

lpnorm<-lmp(data_test[,i]~cbind(ARMA[,i], garchN[,i], garchT[,i], garchG[,i]), p=1.5)

results[i,4]<-accuracy(fitted(lpnorm), data_test[,i])[2]

}, silent=T)

mean(na.omit(results[,1])) average MSFE of the OLS combination scheme

mean(na.omit(results[,2])) average MSFE of the CLS combination scheme

mean(na.omit(results[,3])) average MSFE of the LAD combination scheme

mean(na.omit(results[,4])) average MSFE of the Lp-norm combination scheme

## Appendix 2

**R procedure used for real data application on the single historical series:**

**Load the necessary packages and take from the dataset the historical series “ULTIMO”**

library(tseries)

library(car)

library(timeSeries)

library(forecast)

library(normalp)

library(aTSA)

library(rugarch)

library(ForecastComb)

library(rio)

attach(BTC_USD_Bitfinex_Dati_Storici_2014_2020_1)

ULTIMO<-BTC_USD_Bitfinex_Dati_Storici_2014_2020_1$Ultimo

plot(ULTIMO,type="l")

length(ULTIMO)

**Stationarization of the historical series and descriptive statistics**

adf.test(ULTIMO)

kpss.test(ULTIMO)

l_ULTIMO<-log(ULTIMO)

plot(l_ULTIMO,type="l")

diff_l_ULTIMO<-diff(l_ULTIMO)

plot(diff_l_ULTIMO,type="l")

adf.test(diff_l_ULTIMO)

summary(ULTIMO)

summary(diff_l_ULTIMO)

summary(l_ULTIMO)

var(ULTIMO)

sqrt(var(ULTIMO))

sqrt(var(l_ULTIMO))

sqrt(var(diff_l_ULTIMO))

sd(diff_l_ULTIMO)

qqnorm(diff_l_ULTIMO)

**Splitting training set (80%) and testing set (20%), fit the training set with some models**

x1_train<-diff_l_ULTIMO[1:1632]

x1_test<-diff_l_ULTIMO[1633:2175]

length(x1_test)

x1_test

auto.arima(x1_train,trace=TRUE)

AR2<-arima(x1_train,order = c(2,0,0))

spec1<-ugarchspec(variance.model = list(model = "sGARCH",garchOrder = c(1, 1)), distribution.model = "norm")

spec2<-ugarchspec(variance.model = list(model = "sGARCH", garchOrder = c(1, 1)), distribution.model = "std")

spec3<-ugarchspec(variance.model = list(model = "sGARCH", garchOrder = c(1, 1)),distribution.model = "ged")

fit1<-ugarchfit(spec1, x1_train, out.sample = 543)

fit2<-ugarchfit(spec2, x1_train, out.sample = 543)

fit3<-ugarchfit(spec3, x1_train, out.sample = 543)

**Forecast and MSFE with individual models**

pred1<-forecast(AR2,lead = 543)[,2]

model2<-ugarchforecast(fit1, n.ahead = 543)

model3<-ugarchforecast(fit2, n.ahead = 543)

model4<-ugarchforecast(fit3, n.ahead = 543)

pred2<-model2@forecast$seriesFor

pred3<-model3@forecast$seriesFor

pred4<-model4@forecast$seriesFor

MSFE1<-sqrt(mean((pred1-x1_test)^2))

MSFE2<-sqrt(mean((pred2-x1_test)^2))

MSFE3<-sqrt(mean((pred3-x1_test)^2))

MSFE4<-sqrt(mean((pred4-x1_test)^2))

**Forecast and MSFE with combination methods**

PRED<-cbind(pred1, pred2, pred3, pred4)

COMB<-foreccomb(x1_test, PRED)

OLS<-comb_OLS(COMB)$Accuracy_Train

CLS<-comb_CLS(COMB)$Accuracy_Train

LAD<-comb_LAD(COMB)$Accuracy_Train

lpnorm<-lmp(x1_test~PRED, p=estimatep(x1_train, mean(x1_train)))

LP<-accuracy(fitted(lpnorm), x1_test)

**Q-Q plot, Jarque-Bera test**

qqnorm(fit1@fit$residuals,main="Q-Q plot OLS")

qqnorm(fit3@fit$residuals,main="Q-Q plot GED-GARCH(1,1)")

qqline(fit1@fit$residuals)

qqline(fit3@fit$residuals)

jarque.bera.test(fit1@fit$residuals)

jarque.bera.test(fit3@fit$residuals)

## Appendix B.2

**Data download**

first.date <- as.Date("2010-01-01")

last.date <- as.Date("2020-01-01")

dwjind<-read.csv('http://r-forge.r-project.org/scm/viewvc.php/*checkout*/pkg/fBasics/data/DowJones30.csv?revision=1&root=rmetrics&pathrev=1',sep=';')

tickers<-colnames(dwjind[,-1])

l.out <- BatchGetSymbols(tickers = tickers,

first.date = first.date,

last.date = last.date,

thresh.bad.data = 1,

type.return = "log",

freq.data = "daily")

Data<-unstack(l.out$df.tickers, l.out$df.tickers$ret.adjusted.prices~l.out$df.tickers$ticker)

Data[1,]<-0

**Time series plots**

for (i in 1:ncol(Data)) {

Data[,i]<-as.ts(Data[,i])

}

par(mar = rep(2, 4), mfrow=c(5,5))

for (i in 1:ncol(Data)) {

plot(Data[,i], main=tickers[i], ylab="Returns", xlab="Obs.", col="blue", type="l")

}

par(mar = rep(2, 4), mfrow=c(5,5))

for (i in 1:ncol(Data)) {

qqPlot(Data[,i], main=tickers[i], id=F)

}

**Descriptive statistics and preliminary tests**

unitroot<-vector(mode="numeric", length = ncol(Data))

for (i in 1:ncol(Data)) {

unitroot[i]<-adf.test(Data[,i])$p.value

}

sumstats<-matrix(NA, nrow=ncol(Data), ncol=6)

for (i in 1:ncol(Data)) {

for (j in 1:6) {

sumstats[i,j]<-summary(Data[,i])[j]

}

}

colnames(sumstats)<-c("Min", "1Q", "Median", "Mean", "3Q", "Max")

rownames(sumstats)<-colnames(Data)

xtable(sumstats, digits=4)

**Training and testing datasets**

f<-0.8 # training set length

t<-nrow(Data) # Dataset length

data_train<-Data[1:round(t*f),]

data_test<-Data[(round(t*f)+1):t,]

**Statistical models' forecasts**

spec1<-ugarchspec(variance.model = list(model = "sGARCH",garchOrder = c(1, 1)), mean.model = list(armaOrder = c(1, 0)), distribution.model = "norm")

spec2<-ugarchspec(variance.model = list(model = "sGARCH", garchOrder = c(1, 1)), mean.model = list(armaOrder = c(1, 0)),distribution.model = "std")

spec3<-ugarchspec(variance.model = list(model = "sGARCH", garchOrder = c(1, 1)), mean.model = list(armaOrder = c(1, 0)),distribution.model = "ged")

ARMA<-matrix(NA, nrow=round((1-f)*t), ncol=ncol(Data))

garchN<-matrix(NA, nrow=round((1-f)*t), ncol=ncol(Data))

garchT<-matrix(NA, nrow=round((1-f)*t), ncol=ncol(Data))

garchG<-matrix(NA, nrow=round((1-f)*t), ncol=ncol(Data))

for (i in 1:ncol(Data)) {

ARMA[,i]<-forecast(Arima(data_train[,i], order=c(1,0,1)), h=round((1-f)*t))$mean

garchN[,i]<-ugarchforecast(ugarchfit(spec1, data_train[,i], solver = 'hybrid'), n.ahead = round((1-f)*t))@forecast$seriesFor

garchT[,i]<-ugarchforecast(ugarchfit(spec2, data_train[,i], solver = 'hybrid'), n.ahead = round((1-f)*t))@forecast$seriesFor

garchG[,i]<-ugarchforecast(ugarchfit(spec3, data_train[,i], solver = 'hybrid'), n.ahead = round((1-f)*t))@forecast$seriesFor

}

**Predictive accuracy - Root Mean Square Error (MSFE) results**

MSFE1<-vector(mode="numeric", length=ncol(Data))

MSFE2<-vector(mode="numeric", length=ncol(Data))

MSFE3<-vector(mode="numeric", length=ncol(Data))

MSFE4<-vector(mode="numeric", length=ncol(Data))

for (i in 1:ncol(Data)) {

MSFE1[i]<-sqrt(mean((ARMA[,i]-data_test[,i])^2))

MSFE2[i]<-sqrt(mean((garchN[,i]-data_test[,i])^2))

MSFE3[i]<-sqrt(mean((garchT[,i]-data_test[,i])^2))

MSFE4[i]<-sqrt(mean((garchG[,i]-data_test[,i])^2))

}

results<-cbind(MSFE1, MSFE2, MSFE3, MSFE4)

rownames(results)<-colnames(Data)

colnames(results)<-c("ARMA(1,1)", "GARCH(1,1)", "t-GARCH(1,1)", "GED-GARCH(1,1)")

results

**Forecast combination**

results<-matrix(NA, nrow = ncol(Data), ncol=3)

for (i in 1:ncol(Data)) try({

results[i,1]<-comb_CLS(foreccomb(data_test[,i], cbind(ARMA[,i], garchN[,i], garchT[,i], garchG[,i])))$Accuracy_Train[2]

results[i,2]<-comb_LAD(foreccomb(data_test[,i], cbind(ARMA[,i], garchN[,i], garchT[,i], garchG[,i])))$Accuracy_Train[2]

lpnorm<-lmp(data_test[,i]~cbind(ARMA[,i], garchN[,i], garchT[,i], garchG[,i]), p=1.5)

results[i,3]<-accuracy(fitted(lpnorm), data_test[,i])[2]

}, silent=T)

rownames(results)<-colnames(Data)

colnames(results)<-c("CLS", "LAD", "Lp-norm")

results

## Appendix 3

**MSFE graphs for different regression-based combination approaches:**

See Figs. 6, 7, 8, 9, 10, 11, 12, 13, 14, 15, 16, 17, 18, 19, 20, 21, 22, 23, 24, 25, 26, 27, 28 and 29.
